# Maternal and neonatal immune response to SARS-CoV-2, IgG transplacental transfer and cytokine profile

**DOI:** 10.3389/fimmu.2022.999136

**Published:** 2022-09-27

**Authors:** Rocío Rubio, Ruth Aguilar, Mariona Bustamante, Erica Muñoz, Miquel Vázquez-Santiago, Rebeca Santano, Marta Vidal, Natalia Rodrigo Melero, Daniel Parras, Pau Serra, Pere Santamaria, Carlo Carolis, Luis Izquierdo, Maria Dolores Gómez-Roig, Carlota Dobaño, Gemma Moncunill, Edurne Mazarico

**Affiliations:** ^1^ Barcelona Institute for Global Health, Hospital Clínic - Universitat de Barcelona, Barcelona, Spain; ^2^ Barcelona Institute for Global Health, Spanish Consortium for Research on Epidemiology and Public Health (CIBERESP), Center for Genomic Regulation (CRG), Barcelona Institute of Science and Technology (BIST), Barcelona, Spain; ^3^ Barcelona Center for Maternal-Fetal and Neonatal Medicine (BCNatal), Hospital Sant Joan de Déu and Hospital Clínic, Institut de Recerca Sant Joan de Déu (IR-SJD), Barcelona, Spain; ^4^ Biomolecular screening and Protein Technologies Unit, Centre for Genomic Regulation (CRG), The Barcelona Institute of Science and Technology, Barcelona, Spain; ^5^ Pathogenesis and treatment of autoimmunity department, Institut D'Investigacions Biomèdiques August Pi i Sunyer (IDIBAPS), Barcelona, Spain; ^6^ Julia McFarlane Diabetes Research Centre (JMDRC), and Department of Microbiology, Immunology and Infectious Diseases, Snyder Institute for Chronic Diseases, Cumming School of Medicine, University of Calgary, Calgary, AB, Canada; ^7^ Barcelona Institute for Global Health, CIBER de Enfermedades Infecciosas (CIBERINFEC), Barcelona, Spain

**Keywords:** SARS-CoV-2, maternal and neonatal immunity, antibodies, cytokines, transplacental transfer

## Abstract

SARS-CoV-2 infected pregnant women are at increased risk of severe COVID-19 than non-pregnant women and have a higher risk of adverse pregnancy outcomes like intrauterine/fetal distress and preterm birth. However, little is known about the impact of SARS-CoV-2 infection on maternal and neonatal immunological profiles. In this study, we investigated the inflammatory and humoral responses to SARS-CoV-2 in maternal and cord blood paired samples. Thirty-six pregnant women were recruited at delivery at Hospital Sant Joan de Déu, Barcelona, Spain, between April-August 2020, before having COVID-19 available vaccines. Maternal and pregnancy variables, as well as perinatal outcomes, were recorded in questionnaires. Nasopharyngeal swabs and maternal and cord blood samples were collected for SARS-CoV-2 detection by rRT-PCR and serology, respectively. We measured IgM, IgG and IgA levels to 6 SARS-CoV-2 antigens (spike [S], S1, S2, receptor-binding domain [RBD], nucleocapsid [N] full-length and C-terminus), IgG to N from 4 human coronaviruses (OC43, HKU1, 229E and NL63), and the concentrations of 30 cytokines, chemokines and growth factors by Luminex. Mothers were classified as infected or non-infected based on the rRT-PCR and serology results. Sixty-four % of pregnant women were infected with SARS-CoV-2 (positive by rRT-PCR during the third trimester and/or serology just after delivery). None of the newborns tested positive for rRT-PCR. SARS-CoV-2 infected mothers had increased levels of virus-specific antibodies and several cytokines. Those with symptoms had higher cytokine levels. IFN-α was increased in cord blood from infected mothers, and in cord blood of symptomatic mothers, EGF, FGF, IL-17 and IL-15 were increased, whereas RANTES was decreased. Maternal IgG and cytokine levels showed positive correlations with their counterparts in cord blood. rRT-PCR positive mothers showed lower transfer of SARS-CoV-2-specific IgGs, with a stronger effect when infection was closer to delivery. SARS-CoV-2 infected mothers carrying a male fetus had higher antibody levels and higher EGF, IL-15 and IL-7 concentrations. Our results show that SARS-CoV-2 infection during the third trimester of pregnancy induces a robust antibody and cytokine response at delivery and causes a significant reduction of the SARS-CoV-2-specific IgGs transplacental transfer, with a stronger negative effect when the infection is closer to delivery.

## Introduction

Coronavirus 2019 (COVID-19) pandemic caused by the severe acute respiratory syndrome coronavirus 2 (SARS-CoV-2), has resulted in more than six million deaths and has infected over 500 million people as of July 19, 2022 (1). The most severe outcomes of COVID-19 have been documented in geriatric individuals and pregnant women with chronic diseases, including hypertension, diabetes, and cardiopulmonary problems, or with some other respiratory viral infections (2).

The immune status of pregnant women adapts to ensure tolerance to the fetus by changing the cellular composition and the functions of immune cells. T cell-mediated immunity and humoral responses are suppressed particularly during the third trimester (3), production of IL-4 and IL-10 are increased while IL-2 and IFN-γ are reduced in peripheral blood mononuclear cells (4, 5). As a consequence, pregnant women are at increased risk of morbidity and mortality from respiratory viral infections (6–9). Several studies show that pregnant women with COVID-19 are more likely to be hospitalized and have increased rates of ICU admissions and mechanical ventilation compared with non-pregnant women with COVID-19, resulting in a higher risk of mortality (10–15).

The maternal inflammatory response induced by SARS-CoV-2 infection may also have deleterious effects on the offspring. Although vertical transmission has been hardly observed in SARS-CoV-2 to date (16), with a rate of around 3% (17), pregnant women with COVID-19 are at increased risk of adverse pregnancy outcomes like intrauterine/fetal distress and preterm birth (11, 18). The pro-inflammatory cytokines induced during infection may enter the amniotic cavity and interfere with normal fetal development (19–21) causing short- and long-term damage (22–24) such as miscarriage, restricted fetal growth, or still-birth (25) and neurodevelopmental affection as seen in other viral infections ranging from autism spectrum disorder, attention deficit hyperactivity disorder, and cognitive dysfunction, to anxiety, depression, and schizophrenia (26). Recent studies evidence that SARS-CoV-2 infection during pregnancy results in offspring neurodevelopmental morbidity (26). Maternal infection may also alter the ability of antibodies to transfer across the placenta as has been shown for other infections like HIV (27), malaria (28), dengue (29) or Zika (30) and recently in COVID-19 (31–34). Vaccination of pregnant women with an mRNA COVID-19 vaccine resulted in a significantly greater antibody persistence in infants than infection at 20 to 32 weeks gestation. At 6 months, 57% of infants born to vaccinated mothers had detectable antibodies compared with 8% of infants born to infected mothers (35).

However, our knowledge is limited regarding the immune response in pregnant women infected by SARS-CoV-2 and the immunopathological mechanisms in maternal and neonatal outcomes. Studying the maternal immune response after infection and the *in-utero* antibody transfer can help understand neonatal vulnerability to infection and protection.

In this study, we investigated the inflammatory and humoral responses to SARS-CoV-2 in maternal and cord blood samples collected from pregnant women who had tested positive during the third trimester of pregnancy, and their neonates, by measuring antibody levels against SARS-CoV-2 antigens, their IgG transplacental transfer, and cytokine concentrations.

## Materials and methods

### Study design, participants and sample collection

This is a prospective case-control study in which pregnant women were recruited at delivery at Hospital Sant Joan de Déu (HSJD), Barcelona (Spain) between April and August 2020, before having COVID-19 available vaccines. Women approached were those visited at the hospital, or who arrived in labor or with a programmed induction of labor. To capture the maximum number of SARS-CoV-2 infected mothers inclusion criteria were: i) women with a positive SARS-CoV-2 rRT-PCR; and/or ii) symptoms consistent with SARS-CoV-2 infection (high temperature, fatigue, muscular pain, diarrhea, taste and smell loss, cough, difficulty breathing, pneumonia); and/or iii) direct contact (more than 15 min and less than 2 m distance) with someone diagnosed with COVID-19 by rRT-PCR during the third trimester. Pregnant women who did not meet any of the inclusion criteria were recruited for the control group. Women with multiple gestations, aged <18 or >45 years, with a fetus affected by abnormal karyotype, structural abnormalities, congenital infections, or who did not communicate in Catalan, Spanish or English, were excluded from the study. Thirty-six mother-newborn pairs were enrolled in the study. The study protocol was approved by the Ethics Committee for Clinical Research of the HSJD with the approval code number PIC 48-20, and informed consent was obtained from each participant.

Maternal (age, parity, ethnic group and pathologies) and pregnancy (high blood pressure, gestational diabetes, fetal growth retardation, preterm delivery or other pregnancy complications) variables as well as perinatal outcomes (type of delivery or any newborn complications), were recorded in a questionnaire or obtained from medical records.

Maternal and neonatal nasopharyngeal swabs, and maternal peripheral and cord blood samples, were collected just after delivery. Nasopharyngeal swabs were used for SARS-CoV-2 detection by rRT-PCR. Blood samples were processed to isolate serum that was stored at -80˚C until the analysis of antibody levels and cytokines. Data from the rRT-PCR and serology tests performed with the samples collected just after delivery was used to reallocate mothers to the case (infected) or control (not infected) groups, for the subsequent analyses.

### Antibody luminex assays

IgM, IgG and IgA antibodies to six SARS-CoV-2 antigens were measured in mother and cord serum samples by Luminex as previously described (36). SARS-CoV-2 antigens included in the panel were: the spike full-length protein (S), its subunits S1 and S2, the receptor-binding domain (RBD), the nucleocapsid full-length protein (N FL) and its C-terminus region (N CT) (37). The full-length N proteins from the four HuCoVs OC43, HKU1, 229E and NL63 (37) were also included in the IgG panel. A positive control curve and four blanks were included in each assay plate for QA/QC purposes, and 129 pre-pandemic samples as negative controls to estimate the seropositivity cutoffs. To quantify IgM, test samples and controls were pre-treated with anti-human IgG (Gullsorb) to avoid IgG interferences. Paired mother-cord samples were tested in the same assay plate. At least 50 microspheres per analyte/well were acquired, and the median fluorescence intensity (MFI) was reported for each analyte. Assay positivity cutoffs specific for each isotype and antigen were calculated as 10 to the mean plus 3 standard deviations (SD) of log_10_-transformed MFI of the 129 pre-pandemic controls. A more detailed description of the antigens and the assay are included in the **Supplementary Material**.

### Cytokine luminex assay

The Cytokine Human Magnetic 30-Plex Panel from Invitrogen™ was used to measure the concentrations of 30 analytes in serum from mothers and cord blood samples. Samples were tested using half of the reagents following a modification of the manufacturer’s protocol (38–40). Paired mother-cord samples were tested in the same assay plate. Each plate included 16 serial dilutions (2-fold) of a standard curve, two blank controls and three positive controls of high, medium and low concentrations for QA/QC purposes. Samples were acquired on a Luminex^®^ 100/200. The interpolation of concentrations and imputation of missing data was done using the R package drLumi (41). A more detailed description of the analytes and the assay are included in the **Supplementary Material**.

### Groups definitions and statistical analysis

SARS-CoV-2 infected mother groups were reallocated using serological data from the antibody Luminex assay. Thus, mothers were classified as infected when having a SARS-CoV-2 positive rRT-PCR during the third trimester and/or being seropositive just after delivery, and non-infected when not having reported any SARS-CoV-2 rRT-PCR positive during the third trimester and being seronegative just after delivery. Pregnant women were considered seropositive if positive for one or more isotype-antigen pairs. Time since the infection was estimated as the time since positive rRT-PCR or symptoms onset to delivery. This variable was not available for three mothers who were seropositive just after delivery but did not report any positive rRT-PCR or symptoms.

A comparison of clinical and demographic characteristics between infected and non-infected mothers was performed by the Wilcoxon-rank-sum-test for age (continuous) and by Fisher’s exact test for the rest of the variables (categorical). Antibody and cytokine data were log_10_-transformed to perform the statistical analysis and non-parametric tests were used. The IgG transplacental transfer was calculated as the log_10_-transformed ratio of antibody levels (MFI) in cord vs. mother in paired samples. Comparisons of antibody and cytokine levels between the study groups were performed by the Wilcoxon-rank-sum-test. Correlations between analytes were assessed with Spearman’s rank correlation coefficient ρ (rho) and p-values were computed *via* the asymptotic t approximation. Univariable linear regression models were performed to assess the associations between IgG transplacental transfer (ratio MFI Cord/MFI Mother) as the outcome variable and SARS-CoV-2 infection, infection during the third trimester or just after delivery, time since infection, gestational age, and birth weight as predictor variables. Nominal p-values (not adjusted for multiple testing due to small sample size) of <0.05 were considered statistically significant. All data processing and statistical analyses were performed using the R software version 4.1.1.

## Results

### Pregnancy outcomes in SARS-CoV-2 infected mothers

Our study included 36 pregnant women, 23 SARS-CoV-2 infected and 13 non-infected. No differences were observed between these two groups regarding parity, maternal age at delivery, ethnicity or pathologies (**Table 1**). Among the non-infected women, two reported COVID-19 compatible symptoms probably from another viral infection. Although having symptoms consistent with COVID-19 was an inclusion criterion for those infected or possibly infected with SARS-CoV-2, we included these two cases in the non-infected group because they were neither rRT-PCR positive nor seropositive. Thirty-nine % (9/23) of the infected women showed pregnancy complications vs. 15% (2/13) of the non-infected, although differences were not statistically significant (p=0.086). Pregnancy complications were of different etiologies without a predominant one (**Table 1**). Twenty-two % (5/23) of the newborns from infected women had complications vs. none of the 13 born from non-infected mothers, although differences were not statistically significant (p=0.089). Newborn complications consisted of three premature, one with hyperbilirubinemia and one with lymphopenia. None of the newborns tested positive for SARS-CoV-2 rRT-PCR just after delivery, and neither showed detectable levels of IgM or IgA in cord blood, which are indicative of fetal infection (data not shown).

**Table 1:** Characteristics of the study population.

Variable	All	SARS-CoV-2 Non-infected	SARS-CoV-2 Infected	p-value
N (%)	36	13 (36.1%)	23 (63.9%)	
Maternal age at delivery (median (IQR))	34 (7.5)	33 (5.5)	35 (10)	0.960
Ethnic group N				
African	3 (8.3%)	0	3 (13.1%)	0.473
European	23 (63.9%)	10 (76.9%)	13 (56.5%)	
South American	8 (22.2%)	3 (23.1%)	5 (21.7%)	
South Asiatic	2 (5.6%)	0	2 (8.7%)	
Any maternal pathology* yes	7 (19.4%)	3 (23.1%)	4 (17.4%)	0.686
Parity: Nulliparous	21 (58.3%)	8 (61.5%)	13 (56.5%)	0.385
COVID-19 compatible symptoms				
asymptomatic	23 (63.8%)	11 (84.6%)	12 (52.2%)	0.054
mild	10 (27.7%)	2 (15.5%)	8 (34.8%)	
moderate	1 (2.7%)	0	1 (4.3%)	
severe	2 (5.5%)	0	2 (8.7%)	
Pregnancy complications^†^ yes	11 (30.6%)	2 (15.4%)	9 (39.1%)	0.086
High blood pressure yes	1 (2.8%)	0	1 (4.3%)	0.639
Gestational diabetes yes	4 (11.1%)	1 (7.7%)	3 (13.1%)	0.541
Other pregnancy complications^‡^ yes	6 (16.7%)	0	6 (26.1%)	0.052
Delivery type				
cesarean	15 (41.6%)	4 (30.8%)	11 (47.8%)	0.261
vaginal	21 (58.4%)	9 (69.2%)	12 (52.2%)	
Newborn complications^§^ yes	5 (13.9%)	0	5 (21.7%)	0.089
Preterm delivery yes	4 (11.1%)	1 (7.7%)	3 (13.1%)	0.541
Fetal growth retardation yes	3 (8.3%)	1 (7.7%)	2 (8.7%)	0.708
Low birth weight yes	6 (16.7%)	2 (15.4%)	4 (17.4%)	0.631
Child SARS-CoV-2 rRT-PCR positive	0	0	0	1

^*^N = 3 hypothyroidism; 1 hyperthyroidism; 1 celiac disease; 1 asthma; 1 hypothyroidism and eating disorder.

^†^N = 1 high blood pressure; 4 gestational diabetes; 3 fetal growth retardations; 4 preterm deliveries.

^‡^N = 2 gestational cholestasis; 2 membranes rupture 31.5 weeks and 22 weeks; 1 carbohydrates intolerance; 1 genital herpes.

^§^N = 3 premature; 1 hyperbilirubinemia phototherapy; 1 lymphopenia.

For numerical variables, the median and interquartile range , in brackets, are given. For the categorical variables, the number of individuals for each group and percentages, in parentheses, are given. For the age variable, the Wilcoxon-rank-sum-test was used to compare differences between median values. For the categorical variables, the Fisher exact test was used.

### Antibody response in SARS-CoV-2 infected mothers

**Figure 1:**
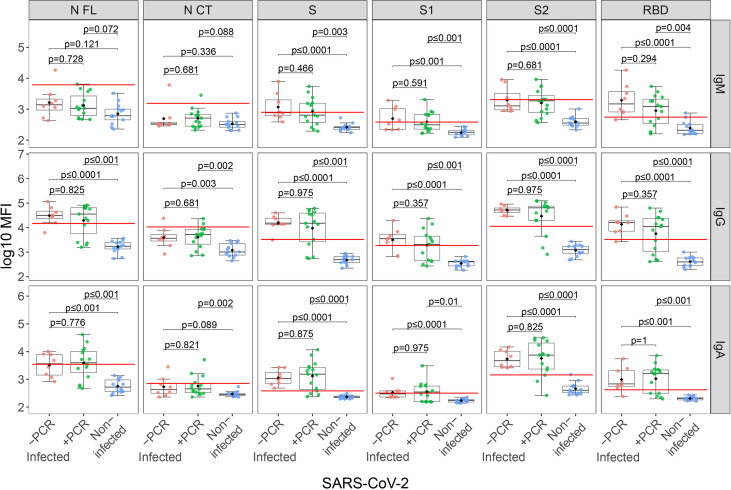
Anti-SARS-CoV-2 antibody levels in mothers stratified by infection and rRT-PCR (PCR) results. Comparison of anti-SARS-CoV-2 IgM, IgG and IgA serum levels (log_10_ median fluorescence intensity, MFI) between infected mothers who tested COVID-19 positive by rRT-PCR during the third trimester (+PCR, N=15, in green), those that tested negative (-PCR, N=8, in red) and non-infected mothers (N=13, in blue). The boxplots represent the median (bold line), the mean (black diamond), the 1^st^ and 3^rd^ quartiles (box) and the largest and smallest values within 1.5 times the inter-quartile range (whiskers). Groups were compared by the Wilcoxon-rank-sum-test. The red line indicates the seropositivity cutoff calculated as 10 to the mean plus 3 standard deviations (SD) of log_10_-transformed MFI of 129 pre-pandemic controls. Antigens: nucleocapsid full-length (N FL) and C-terminus (N CT), spike full-length (S), S1 and S2 subunits, and receptor-binding domain (RBD).

From the infected group (N=23), 11 were rRT-PCR positive in the third trimester and seropositive just after delivery; eight did not report any rRT-PCR positive during pregnancy but were seropositive just after delivery (5 reported symptoms during the third trimester and 3 were asymptomatic), and 4 were rRT-PCR positive but seronegative just after delivery, denoting recent infections or deficiency in producing antibodies. Anti-SARS-CoV-2 antibody levels did not differ between infected rRT-PCR positive (N=15) and negative (N=8) mothers during the third trimester (**Figure 1**). Infected mothers showed higher antibody levels compared to non-infected mothers (p≤0.01) regardless of the rRT-PCR status, except for IgM against N antigen (**Figure 1**). Among the infected mothers, those with symptoms showed an overall trend of higher antibody levels compared to asymptomatic mothers although not statistically significant except for IgA to RBD (p=0.049) (**Figure S1**).

### Effect of time since infection on SARS-CoV-2 antibody levels

**Figure 2:**
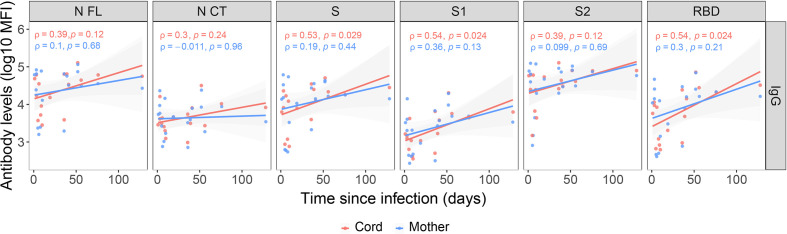
Correlations between anti-SARS-CoV-2 IgG levels and time since infection. Correlations of anti-SARS-CoV-2 IgG levels (log_10_ median fluorescence intensity, MFI) in infected mothers (blue) and cord blood (red) with time since infection (days since positive rRT-PCR or symptoms onset), represented as a linear model with standard error as confidence interval (shaded areas) and assessed by the Spearman test, showing the rho (ρ) and p-values. Only data from SARS-CoV-2 infected mothers and their newborns are included (N=20). Antigens: nucleocapsid full-length (N FL) and C-terminus (N CT), spike full-length (S), S1 and S2 subunits, and receptor-binding domain (RBD).

We evaluated the association of antibody levels with time since infection in paired infected mother-cord samples. Anti-SARS-CoV-2 IgG levels in cord blood showed moderate positive associations with time since infection (ρ=0.3-0.54) for all antigens, being lower when the infection was closer to delivery, as expected, and the associations were statistically significant for S, S1 and RBD (**Figure 2**). However, only IgG levels to S1 and RBD showed moderate positive associations with time since infection in maternal samples (ρ=0.3) and were not statistically significant (**Figure 2**).

### IgG transplacental transfer in SARS-CoV-2 infected mothers

**Figure 3:**
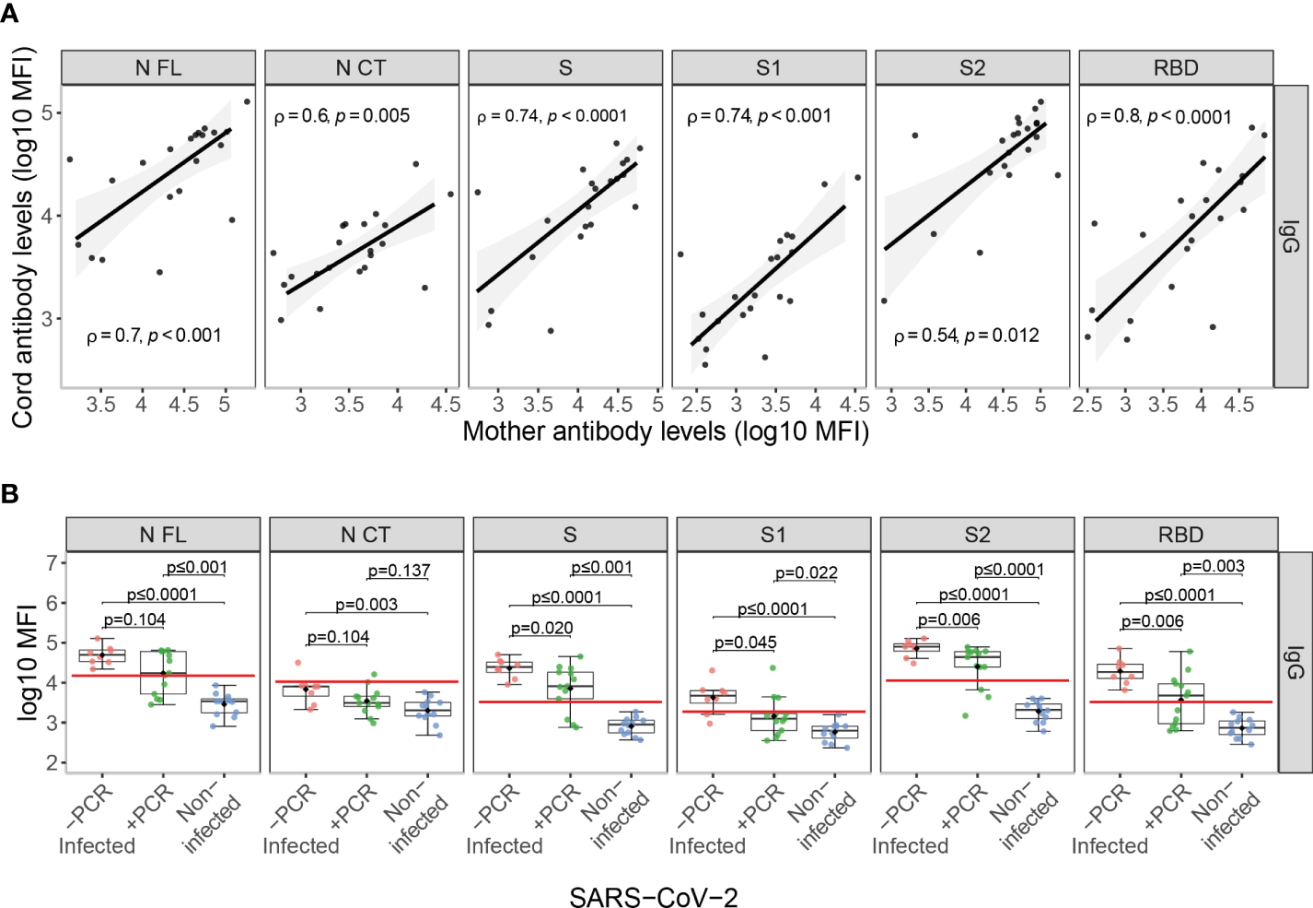
Anti-SARS-CoV-2 antibody levels in cord blood. **(A)** Correlations of anti-SARS-CoV-2 IgG levels (log_10_ median fluorescence intensity, MFI) in paired infected mothers and cord blood samples represented as a linear model with standard error as confidence interval (shaded areas) and assessed by the Spearman test, showing the rho (ρ) and p-values. **(B)** Comparison of anti-SARS-CoV-2 IgG cord blood levels (log_10_ MFI) between infected mothers who tested COVID-19 rRT-PCR positive (+PCR, N=13, in green) during the third trimester, those that tested rRT-PCR negative (-PCR, N=8, in red) and from non-infected mothers (N=12, in blue). The boxplots represent the median (bold line), the mean (black diamond), the 1^st^ and 3^rd^ quartiles (box) and the largest and smallest values within 1.5 times the inter-quartile range (whiskers). Groups were compared by the Wilcoxon-rank-sum-test. The red line indicates the seropositivity cutoff calculated as 10 to the mean plus 3 standard deviations (SD) of log_10_-transformed MFI of 129 pre-pandemic controls. Antigens: nucleocapsid full-length (N FL) and C-terminus (N CT), spike full-length (S), S1 and S2 subunits, and receptor-binding domain (RBD).

**Figure 4:**
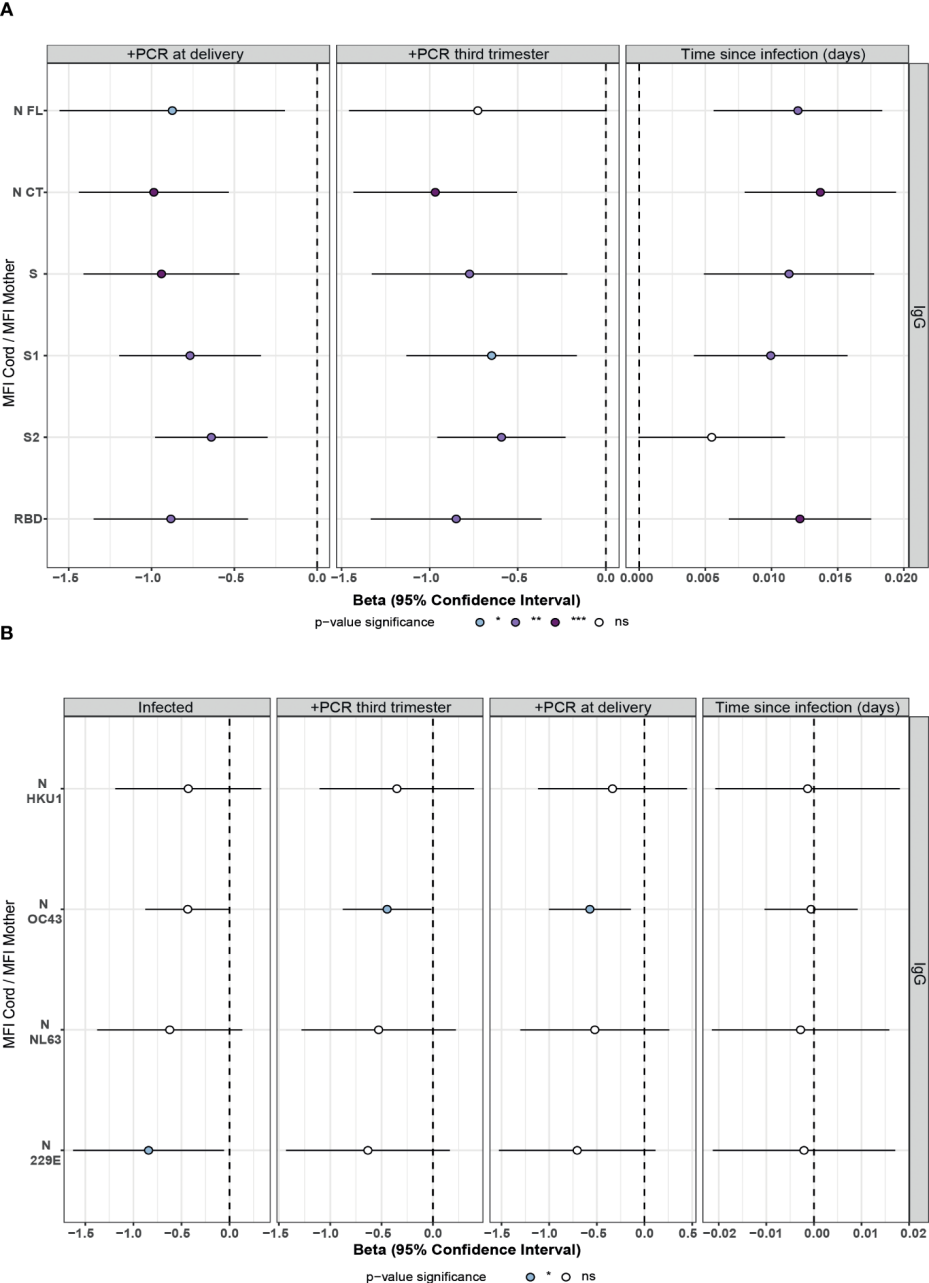
Association of SARS-CoV-2 infection and time since infection on IgG transfer from mother to the newborn in univariable linear regression models. Forest plots show the effect of SARS-CoV-2 infection, positive rRT-PCR (+PCR) during the third trimester or at delivery, and the time since infection (days since positive rRT-PCR or symptoms onset) on IgG transfer from mother to the newborn (MFI Cord/MFI Mother) for **(A)** SARS-CoV-2 antigens in seropositive paired infected mothers and cord blood and **(B)** HuCoVs N proteins in all study population. Univariable linear regression models were fitted to calculate the betas (dots) and 95% confidence intervals (CI) (lines). The color of the dots represents the p-value significance, where *p-value ≤ 0.05, **p-value ≤ 0.01 and ***p-value ≤ 0.001, ns, not significant. Antigens: nucleocapsid full-length (N FL) and C-terminus (N CT), spike full-length (S), S1 and S2 subunits, and receptor-binding domain (RBD).

Among infected mothers, antigen-specific IgG levels in serum showed moderate to strong positive correlations with levels in cord blood (ρ=0.54-0.8, p<0.05, **Figure 3A**). Contrary to what was observed in mother samples, IgG levels to S antigens in cord blood showed significant differences depending on the the maternal rRT-PCR status during the third trimester (p<0.05), being higher in the infected but rRT-PCR negative (**Figure 3B**), suggesting a negative effect of infection during the third trimester in the IgG transfer. To confirm this observation, we assessed the effect of the SARS-CoV-2 infection on the IgG transplacental transfer by linear regression models in paired seropositive infected mothers and cord blood. The rRT-PCR positive mothers just after the delivery or during the third trimester transferred fewer anti-SARS-CoV-2 IgGs to cord blood compared to rRT-PCR negative, except for N FL antigen when tested positive during the third trimester (**Figures 4A** and **S2A**, p<0.01). Then, we investigated if this IgG transplacental transfer reduction was extensible to HuCoVs antigens by linear regression models in all study population. Being infected with SARS-CoV-2 reduced the transference of IgG to 229E N antigen (p<0.05) and having a positive rRT-PCR, either during the third trimester or just after delivery, to OC43 N antigen (p<0.05) (**Figures 4B** and **S2B**). This suggests a most noticeable negative effect for anti-SARS-CoV-2 antibodies. Gestational age and birth weight, two variables strongly associated with antibody transfer, did not affect the SARS-CoV-2 IgG transplacental transfer in seropositive infected mothers (**Figure S3**).

According to the lower anti-SARS-CoV-2 IgG levels observed in cord blood in recent compared to past infections (**Figure 2**), there was a positive effect of time since infection on anti-SARS-CoV-2 IgG transplacental transfer (**Figures 4A** and **S4**). This observation together with the lack of correlation of maternal antibody levels with time since infection, confirms the impact of SARS-CoV-2 infection on IgG transplacental transfer and suggests a stronger negative effect when the infection is closer to delivery. Time since infection did not affect the HuCoVs IgG transference (**Figure 4B**).

### Fetus sex effect on SARS-CoV-2 antibody response

**Figure 5:**
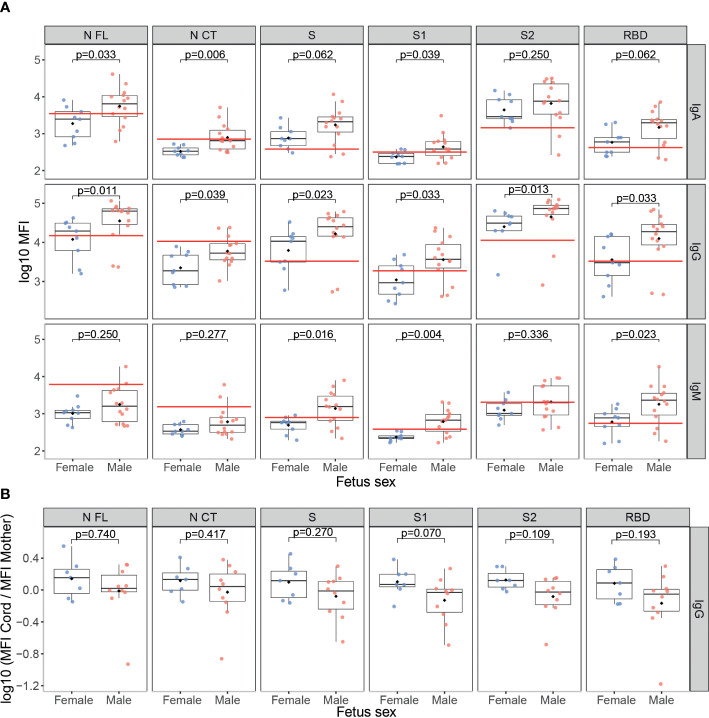
Anti-SARS-CoV-2 antibody levels by fetus sex. **(A)** Comparison of anti-SARS-CoV-2 levels (log_10_ median fluorescence intensity, MFI) in infected mothers between those who had a male fetus (N=14, in red) and those who had a female fetus (N=9, in blue). **(B)** Comparison of the ratios of IgG levels (log_10_ MFI) to SARS-CoV-2 antigens in cord blood vs. mother peripheral blood from seropositive mothers who had a male fetus (N=10, in red) and those that had a female fetus (N=7, in blue). The boxplots represent the median (bold line), the mean (black diamond), the 1^st^ and 3^rd^ quartiles (box) and the largest and smallest values within 1.5 times the inter-quartile range (whiskers). Groups were compared by the Wilcoxon-rank-sum-test. The red line indicates the seropositivity cutoff calculated as 10 to the mean plus 3 standard deviations (SD) of log_10_-transformed MFI of 129 pre-pandemic controls. Antigens: nucleocapsid full-length (N FL) and C-terminus (N CT), spike full-length (S), S1 and S2 subunits, and receptor-binding domain (RBD).

SARS-CoV-2 infected mothers with a male fetus had higher SARS-CoV-2 antibody levels in serum compared to those with a female fetus (**Figure 5A**), specifically IgM against S, S1 and RBD (p<0.05); IgA against N antigens and S1 (p<0.05); and IgG to all antigens (p<0.05). However, no differences were found in cord blood samples (data not shown). Also, no differences were found by fetus sex in antibody levels against the N antigen of the HuCoVs, neither in the mother nor cord blood (data not shown). Interestingly, there was a trend of lower anti-SARS-CoV-2 IgG transplacental transfer in mothers carrying a male fetus compared to mothers carrying a female (**Figure 5B**), but not statistically significant. The same trend, not statistically significant, by fetus sex was observed for the transfer of IgG to N of the HuCoVs in SARS-CoV-2 infected mothers (**Figure S5**).

### Cytokine profile in SARS-CoV-2 infected mothers

**Figure 6:**
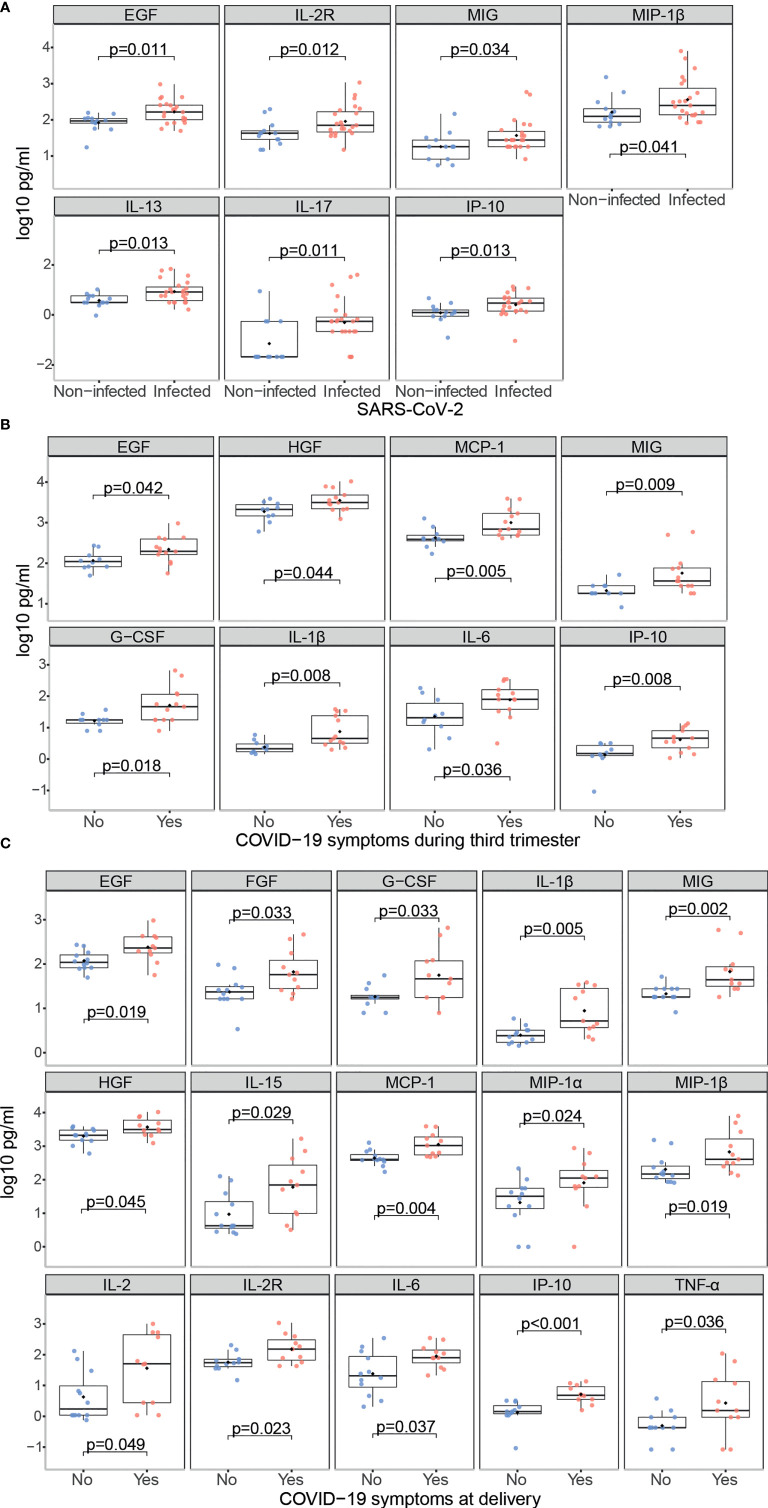
Cytokine, chemokine and growth factor concentrations in SARS-CoV-2 infected mothers. Comparison of inflammatory markers concentrations (log_10_ pg/ml) in serum between **(A)** Infected (N=23, in red) vs. non-infected (N=13, in blue) mothers; **(B)** Infected symptomatic (N=13, in red) vs. asymptomatic (N=10, in blue) mothers during the third trimester; **(C)** Infected symptomatic (N=11, in red) vs. asymptomatic (N=12, in blue) mothers at delivery. Groups were compared by the Wilcoxon-rank-sum-test. Only significant comparisons are shown, the remaining are in **Figures S6–8**.

SARS-CoV-2 infected mothers had higher serum concentrations of the growth factor EGF, the Th2 cytokine IL-13, the pro-inflammatory marker IL-2R, the anti-inflammatory cytokine IL-17, and the chemokines IP-10, MIG and MIP-1β (p<0.05) compared to non-infected mothers (**Figures 6A** and **S6**). Those with symptoms at any time during the third trimester showed higher levels of growth factors EGF, G-CSF and HGF, the pro-inflammatory cytokines IL-1β and IL-6, and the chemokines IP-10, MCP-1 and MIG (p<0.05) compared to the asymptomatic ones (**Figures 6B** and **S7**); and when the symptoms were at delivery, FGF, the anti-inflammatory cytokine IL-15, the Th1 markers IL-2, IL-2R, MIP-1α, MIP-1β and the pro-inflammatory cytokine TNF-α (p<0.05) were also increased (**Figures 6C** and **S8**).

**Figure 7:**
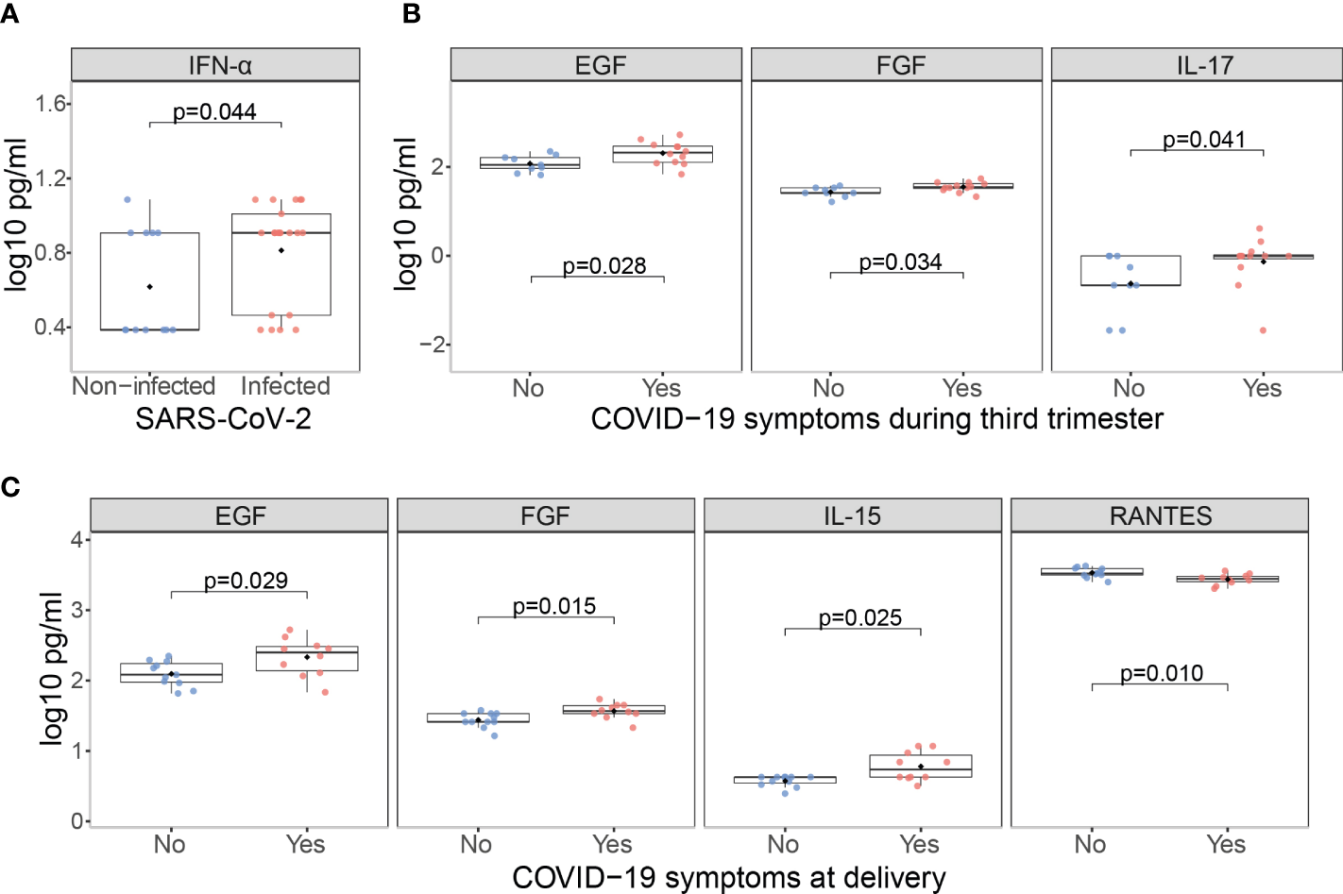
Cytokine, chemokine and growth factor concentrations in cord blood from children born from SARS-CoV-2 infected mothers. Comparison of inflammatory markers concentrations (log_10_ pg/ml) in cord blood between **(A)** Infected (N=21, in red) vs. non-infected (N=12, in blue) mothers; **(B)** Infected symptomatic (N=12, in red) vs. asymptomatic (N=9, in blue) mothers during the third trimester; **(C)** Infected symptomatic (N=11, in red) vs. asymptomatic (N=12, in blue) mothers at delivery. Groups were compared by the Wilcoxon-rank-sum-test. Only significant comparisons are shown, the remaining are in **Figures S9–11**.

**Figure 8:**
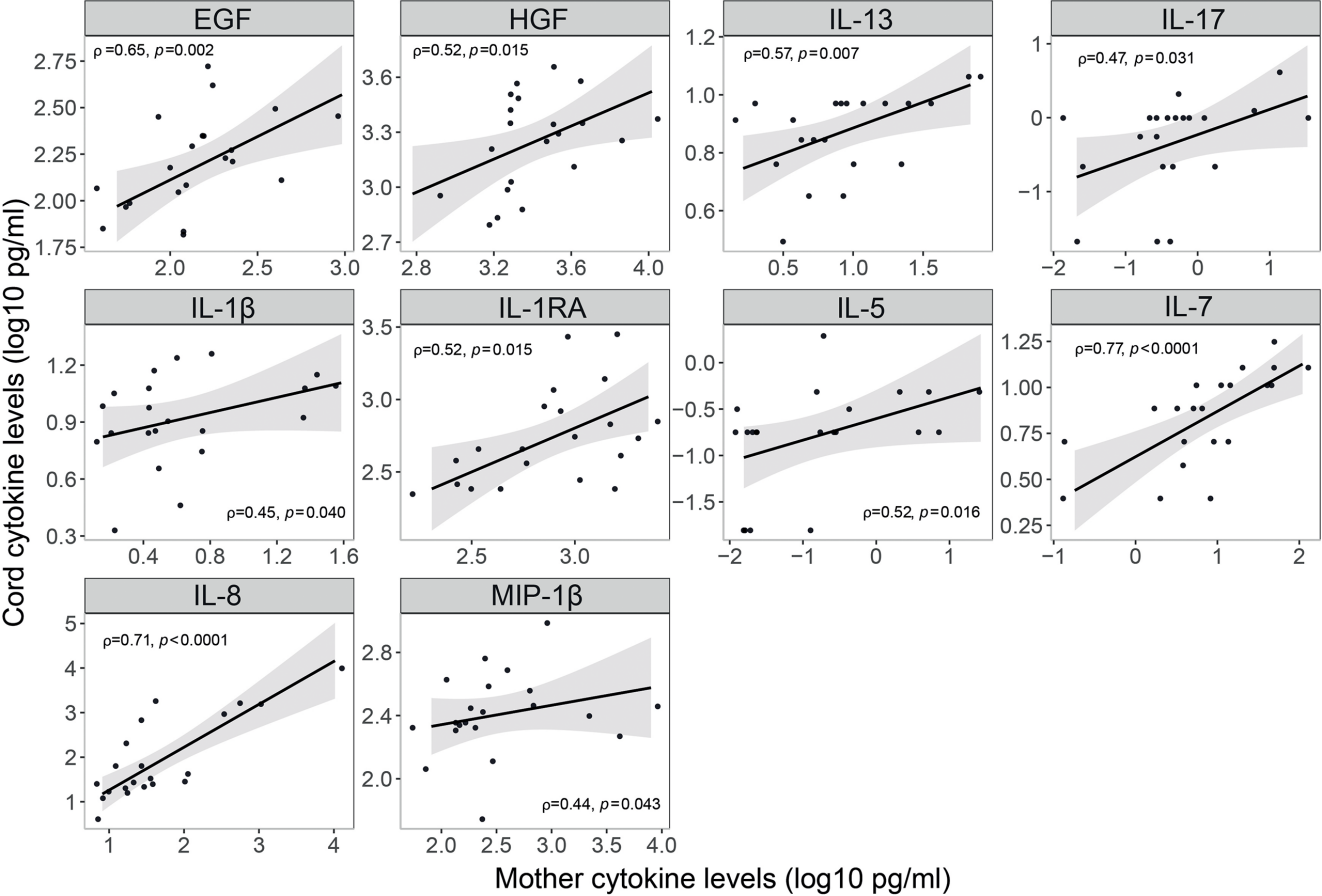
Correlations of cytokine, chemokine and growth factor concentrations in serum and cord blood from SARS-CoV-2 infected mothers. Correlations of inflammatory markers concentrations (log_10_ pg/ml) in serum blood and cord blood from SARS-CoV-2 infected mothers are represented as a linear model with standard error as a confidence interval (shaded areas). Correlations were assessed by the Spearman test, and the rho (ρ) and p-values are shown. Only significant correlations are shown, the remaining are in **Figure S12**.

Newborns from infected mothers showed higher concentrations of the pro-inflammatory cytokine IFN-α (p=0.044) in cord blood than those from non-infected mothers (**Figures 7A** and **S9**). Newborns from mothers with symptoms at any time during the third trimester presented higher levels of EGF, FGF and IL-17 (p<0.05) in cord blood compared to those from asymptomatic ones (**Figures 7B** and **S10**); and when symptoms were at delivery IL-15 was also increased while the chemokine RANTES was decreased (p<0.05) (**Figures 7C** and **S11**). Infected mothers showed positive significant correlations for EGF, HGF, IL-13, IL-17, IL-1β, IL-1RA, IL-5, IL-7 and IL-8, and MIP-1β (ρ=0.44-0.77, p<0.05) concentrations in serum and cord blood (**Figures 8** and **S12**).

### Fetus sex effect on SARS-CoV-2 cytokine response

SARS-CoV-2 infected women carrying a male fetus had higher concentrations of EGF, IL-15 and the regulatory cytokine IL-7 in serum compared to women carrying a female fetus (**Figure S13**, p<0.05). There were no differences in any of the analytes tested by the sex of the fetus in cord samples (data not shown).

## Discussion

The SARS-CoV-2 infection during the third trimester induced a robust antibody and cytokine response detected at delivery in the mother and cord blood. Symptomatic mothers induced higher levels of cytokines and SARS-CoV-2 specific antibodies than asymptomatic women, and maternal levels correlated with cord blood levels. However, the infection during the third trimester caused an impairment of the IgG transplacental transfer that was stronger when the infection was closer to delivery. Furthermore, we observed that mothers carrying a male fetus induced higher anti-SARS-CoV-2 antibody levels and had higher EGF, IL-15 and IL-7 concentrations than mothers carrying a female fetus.

Among the infected pregnant women, those with symptoms had higher anti-SARS-CoV-2 antibody levels, especially IgA and IgM to RBD. Higher RBD-IgA levels in symptomatic infections in non-pregnant adults have been previously reported (42–44). Two recent studies have also reported higher RBD-IgG levels at delivery in pregnant patients with symptomatic infections compared to the asymptomatic ones (31, 45), and it probably impacts the passive transfer of protective IgA through breast milk.

SARS-CoV-2 infected mothers with symptoms presented increased levels of several cytokines compared to the asymptomatic ones, and cytokine serum levels correlated with cord blood levels. In a SARS-CoV-2 infection, inflammatory cytokine storms are an important cause of severe manifestations of COVID-19. This is critically important in the case of pregnant women because of the consequences for the fetus. We observed higher levels of IL-1β, IL-6 and TNF in symptomatic mothers, and of IFN-α in cord blood from SARS-CoV-2 infected mothers. Elevated levels of these cytokines may affect fetal development, increase the risk of neurological diseases, and lead to abortion in the first and second trimesters and preterm birth in the third trimester (46). We also observed increased levels of IL-17 in cord blood from infected symptomatic mothers. High levels of IL-17 can lead to cortical dysplasia and behavioral abnormalities in fetuses and increase the probability that offspring develop mental illness in adulthood (46). However, cytokine data have to be considered with caution due to the dramatic fluctuation of cytokines intrapartum. Normal delivery is pro-inflammatory and women with pregnancy complications, such as preterm delivery or fetal growth retardation, have an ever more pro-inflammatory profile (47), making it difficult to disentangle what is due to COVID-19 or to inflammatory changes in the labor process.

We observed that maternal antibody levels highly correlated to cord blood levels, similar to previous studies (31, 45). However, rRT-PCR positive mothers during the third trimester of pregnancy had a reduction in the IgG transplacental transfer, also observed in previous studies (31–34). We also found that the negative effect of the infection on the IgG transfer was stronger as the infection was closer to delivery. Previous studies have reported that the IgG transfer ratio at birth is significantly lower for third trimester as compared to second-trimester infections (48–50). This could be because the mother has been seropositive for a shorter period, therefore maternal antibody levels may have not reached the peak response and there has been less time for transfer of antibodies. However, we did not detect an effect of antibody levels in the mother by time since infection. Moreover, in the transfer ratio, the IgG levels in cord blood we corrected by the IgG levels in the mother. In addition, although it has been previously documented that preterm newborns have reduced IgG transplacental transfer (51, 52), prematurity was ruled out as the cause of the reduction of SARS-CoV-2 IgG transfer because neither gestational age nor birth weight were associated. All together suggest that SARS-CoV-2 infections closer to term have a stronger negative impact on the IgG transplacental transfer. Interestingly, the reduction in IgG transfer was more marked for the anti-SARS-CoV-2 IgGs than for the anti-HuCoV IgGs. This stronger effect on the transfer of anti-SARS-CoV-2 IgGs vs. other IgGs has also been observed in the studies by Edlow et al. (34) and Atyeo et al. (49). They suggest a potential alteration in the Fc region glycosylation of SARS-CoV-2–specific antibodies probably mediated by the inflammation (34, 49), which may result in a compromised transfer by the Fc receptors (53). Neonatal transferred anti-SARS-CoV-2 antibodies may also be of lower activity (49) and short-lived (54), which raises further concern.

Mothers carrying a male fetus had higher concentrations of EGF, IL-15 and IL-7 cytokines, and induced higher antibody levels against SARS-CoV-2 than those carrying a female, but IgG transfer to the fetus was less efficient in the first ones. Previous studies have shown that fetal sex influences maternal serum cytokine and antibody profiles (55–57). In the context of SARS-CoV-2 infection, a recent study has reported similar results to ours, and a more pronounced SARS-CoV-2-specific antibody glycosylation in mothers carrying a male fetus, which would explain the reduced transplacental transfer (58).

In line with previous studies, SARS-CoV-2 infected pregnant women and their newborns showed more gestational complications than the non-infected women, although differences did not reach statistical significance. An increased risk of adverse birth outcomes, preterm birth, preeclampsia, cesarean section, and even stillbirth, neonatal death and maternal mortality has been reported in COVID-19 positive pregnant women (59–63). A surveillance study by the CDC also showed that pregnant women suffer a greater clinical burden due to COVID-19 than their non-pregnant counterparts (10). A meta-analysis of 86 studies reported a high proportion of pregnant women with COVID-19 who had preterm birth (22%) and cesarean delivery (48%), and higher estimated rates of admission to the ICU (7% vs. 4% of non-pregnant women) (64).

One of the main strengths of the study is the broad panel of antigen-specific antibodies of three isotypes, including SARS-CoV-2 and HuCoVs, and cytokines measured, which allows having a very comprehensive picture of the humoral immune response in SARS-CoV-2 infected pregnant women and their newborns. However, the study has some limitations. The main one is the small sample size. Also, we did not adjust the p-values for multiple comparisons due to the small sample size, and therefore results should be taken with caution and require further validation. Larger sample size would have increased the statistical power to detect additional differences. It would have also been interesting to have histological data on placental samples to assess associations with the humoral response, the antibody transfer, and the delivery outcomes.

Results from this study indicate that we should pay greater attention to women infected with SARS-CoV-2 and their newborns. Children born to mothers with SARS-CoV-2 during pregnancy should be longitudinally observed to assess long-term outcomes. In addition, the vaccination of pregnant women should be highly recommended. Safety data on COVID-19 vaccines in pregnancy are rapidly accumulating without safety concerns being detected (65–68). However, additional longitudinal studies of vaccinated pregnant women at different trimesters and information on vaccine breakthroughs are necessary to inform maternally, pregnancy, and infant outcomes.

Future studies should further address the impact of SARS-CoV-2 infection in vaccinated and unvaccinated mothers along with gestational age on immune responses, how those affect fetal development, antibody mammary transfer, and the mechanisms underlying them. This knowledge may provide insights for vaccination strategies to assure the greatest protection for both mother and neonate.

## Data availability statement

The raw data supporting the conclusions of this article will be made available by the authors upon request.

## Ethics statement

The study was reviewed and approved by the Ethics Committee for Clinical Research of the Hospital Sant Joan de Déu. The patients/participants provided their written informed consent to participate in this study.

## Author contributions

EMa, GM, and CD designed the study. EMu processed the samples at HSJD. MB managed the database. RR and RA developed and performed the serological and cytokines assays, the analysis and interpretation of the results. MV-S, RS, and MV gave support with the assays and analysis. LI, CC, PSa, PSe, DP, and NM produced the antigens. GM and CD supervised the assays and data analyses. RA and RR wrote the draft of the manuscript and GM, CD, MG-R, MB, and EMa reviewed the manuscript. All authors contributed to the article and approved the submitted version.

## Funding

This work was supported by the Fundació Privada Daniel Bravo Andreu. RR had the support of the Health Department, Catalan Government (PERIS SLT017/20/000224). LI work was supported by the PID2019-110810RB-I00 grant from the Spanish Ministry of Science & Innovation. We acknowledge support from the Spanish Ministry of Science and Innovation and State Research Agency through the “Centro de Excelencia Severo Ochoa 2019-2023” Program (CEX2018-000806-S), and support from the Generalitat de Catalunya through the CERCA Program. EMa and MG-R work was supported by Instituto de Salud Carlos III (ISCIII), RD21/0012/0003, co-funded by the European Union (NextGenerationEU/PRTR).

## Acknowledgments

We are grateful to the participants of the study. Special thanks to the team members from ISGlobal, specifically Laura Puyol for the organization and coordination of shipment of samples and materials, Diana Barrios and Alfons Jiménez for the technical support in the lab, Gemma Ruiz-Olalla for the statistical support and Jordi Chi for the production of N antigens.

## Conflict of interest

The authors declare that the research was conducted in the absence of any commercial or financial relationships that could be construed as a potential conflict of interest.

## Correction note

A correction has been made to this article. Details can be found at: 10.3389/fimmu.2025.1672095.

## Publisher’s note

All claims expressed in this article are solely those of the authors and do not necessarily represent those of their affiliated organizations, or those of the publisher, the editors and the reviewers. Any product that may be evaluated in this article, or claim that may be made by its manufacturer, is not guaranteed or endorsed by the publisher.
