# Correction: Maternal and neonatal immune response to SARS-CoV-2, IgG transplacental transfer and cytokine profile

**DOI:** 10.3389/fimmu.2025.1672095

**Published:** 2025-11-14

**Authors:** Rocío Rubio, Ruth Aguilar, Mariona Bustamante, Erica Muñoz, Miquel Vázquez-Santiago, Rebeca Santano, Marta Vidal, Natalia Rodrigo Melero, Daniel Parras, Pau Serra, Pere Santamaria, Carlo Carolis, Luis Izquierdo, Maria Dolores Gómez-Roig, Carlota Dobaño, Gemma Moncunill, Edurne Mazarico

**Affiliations:** 1 Barcelona Institute for Global Health, Hospital Clínic - Universitat de Barcelona, Barcelona, Spain; 2 Barcelona Institute for Global Health, Spanish Consortium for Research on Epidemiology and Public Health (CIBERESP), Center for Genomic Regulation (CRG), Barcelona Institute of Science and Technology (BIST), Barcelona, Spain; 3 Barcelona Center for Maternal-Fetal and Neonatal Medicine (BCNatal), Hospital Sant Joan de Déu and Hospital Clínic, Institut de Recerca Sant Joan de Déu (IR-SJD), Barcelona, Spain; 4 Biomolecular screening and Protein Technologies Unit, Centre for Genomic Regulation (CRG), The Barcelona Institute of Science and Technology, Barcelona, Spain; 5 Pathogenesis and treatment of autoimmunity department, Institut D’Investigacions Biomèdiques August Pi i Sunyer (IDIBAPS), Barcelona, Spain; 6 Julia McFarlane Diabetes Research Centre (JMDRC), and Department of Microbiology, Immunology and Infectious Diseases, Snyder Institute for Chronic Diseases, Cumming School of Medicine, University of Calgary, Calgary, AB, Canada; 7 Barcelona Institute for Global Health, CIBER de Enfermedades Infecciosas (CIBERINFEC), Barcelona, Spain

**Keywords:** SARS-CoV-2, maternal and neonatal immunity, antibodies, cytokines, transplacental transfer

The funder “Instituto de Salud Carlos III (ISCIII), RD21/0012/0003” and the “European Union (NextGenerationEU/PRTR)”, to “EMa and MG-R” was erroneously omitted.

The original version of this article has been updated.

