# Supplementary material for: Maternal and neonatal immune response to SARS-CoV-2, IgG transplacental transfer and cytokine profile
Source: Front Immunol. 2022 Sep 27;13:999136. doi: 10.3389/fimmu.2022.999136 (PMC9552073; doi:10.3389/fimmu.2022.999136)
Supplement: Supplementary file 1 [file DataSheet_1.docx]

Supplementary Material

# Supplementary Data

Antibody Luminex assays

IgM, IgG and IgA antibodies to several antigens of SARS-CoV-2 and IgG to antigens from 4 human coronaviruses (HuCoVs) were measured by Luminex following the protocol explained elsewhere(1). The SARS-CoV-2 antigens included in the panel were the spike full protein (S) (aa 1-1213 expressed in Expi293 and His tag-purified), its subregion S1 (aa 1-681, expressed in Expi293 and His tag-purified), both produced at the Center for Genomic Regulation, the subregion S2 (SinoBiological, cat no 40590-V08B), the receptor-binding domain (RBD) (StrepTag purified from the supernatant of lentiviral-transduced CHO-S cells cultured under a fed-batch system), the nucleocapsid full protein (N) and its specific C-terminal region (aa 340-416) (both expressed in *E. coli* and His tag-purified)(2). For the measurement of IgG, we also included the N protein of the following HuCoVs: OC43, HKU1, 229E, and NL63 (all expressed in *E. coli* and His tag-purified)(2). A hyperimmune pool was used as a positive control in each plate assay for QA/QC purposes and was prepared at 2-fold, 8 serial dilutions from 1:500 and 129 pre-pandemic samples were used as negative controls to estimate the seropositivity cutoffs. We also included technical blanks consisting of Luminex Buffer (1% BSA, 0.05% Tween-20, 0.05% sodium azide in PBS) to detect non-specific signals. To quantify IgM responses, test samples and controls were pre-treated with anti-human IgG (Gullsorb) at 1:10 dilution, to avoid IgG interferences. Paired mother-cord samples were tested in the same assay plate. Briefly, multiplexed antigen-coupled microspheres (2000 per analyte per well) were incubated with controls, technical blanks, and test samples with a final sample dilution of 1:500 for one hour to allow specific binding of antibodies. After several washes, phycoerythrin-conjugated goat anti-human IgG (GTIG-001, Moss Bio), IgA (GTIA-001, Moss Bio) or IgM (GTIM-001, Moss Bio) diluted at 1:400, 1:200 and 1:200, respectively, were added and incubated for 30 min to allow antibody binding detection. Finally, after several washes, microspheres were resuspended with Luminex Buffer before acquisition on the Flexmap 3D®. At least 50 microspheres per analyte/well were acquired, and the median fluorescence intensity (MFI) was reported for each analyte. Assay positivity cutoffs specific for each isotype and antigen were calculated as 10 to the mean plus 3 standard deviations (SD) of log_10_-transformed MFI of the 129 pre-pandemic controls.

Cytokine Luminex assay

The Cytokine Human Magnetic 30-Plex Panel from Invitrogen^TM^ was used to measure the concentrations of the following analytes in serum from mothers and cord blood samples: epidermal growth factor (EGF), fibroblast growth factor (FGF), granulocyte colony-stimulating factor (G-CSF), granulocyte-macrophage colony-stimulating factor (GM-CSF), hepatocyte growth factor (HGF), vascular endothelial growth factor (VEGF), tumor necrosis factor (TNF), interferon (IFN)-α, IFN-γ, interleukin (IL)-1RA, IL-1β, IL-2, IL-2R, IL-4, IL-5, IL-6, IL-7, IL-8, IL-10, IL-12(p40/p70), IL-13, IL-15, IL-17, IFN-γ induced protein (IP-10), monocyte chemoattractant protein (MCP-1), monokine induced by IFN-γ (MIG), macrophage inflammatory protein (MIP)-1α, MIP-1β, regulated on activation normal T cell expressed and secreted (RANTES) and eotaxin. Samples were tested in single replicates following a modification of the manufacturer’s protocol explained elsewhere(3–5) Paired mother-cord samples were tested in the same assay plate. Each plate included 16 serial dilutions (2-fold) of a standard sample provided by the vendor, two blank controls and three positive controls of high, medium and low concentrations in duplicate prepared from a reference sample for QA/QC purposes. Samples were acquired on a Luminex® 100/200. The concentration of each analyte was obtained by interpolating the MFI to a 5-parameter logistic regression curve and reported as pg/mL using the drLumi R package(6). Limits of quantification (LOQ) were estimated based on cutoff values of the 30% coefficient of variation (CV) of the standard curve for each analyte. Values below the lower LOQ (LLOQ) were assigned a random value between the LLOQ and the LLOQ/2. Values above the upper LOQ (ULOQ) were assigned a random value between the ULOQ and the ULOQx2.

# Supplementary Figures

**
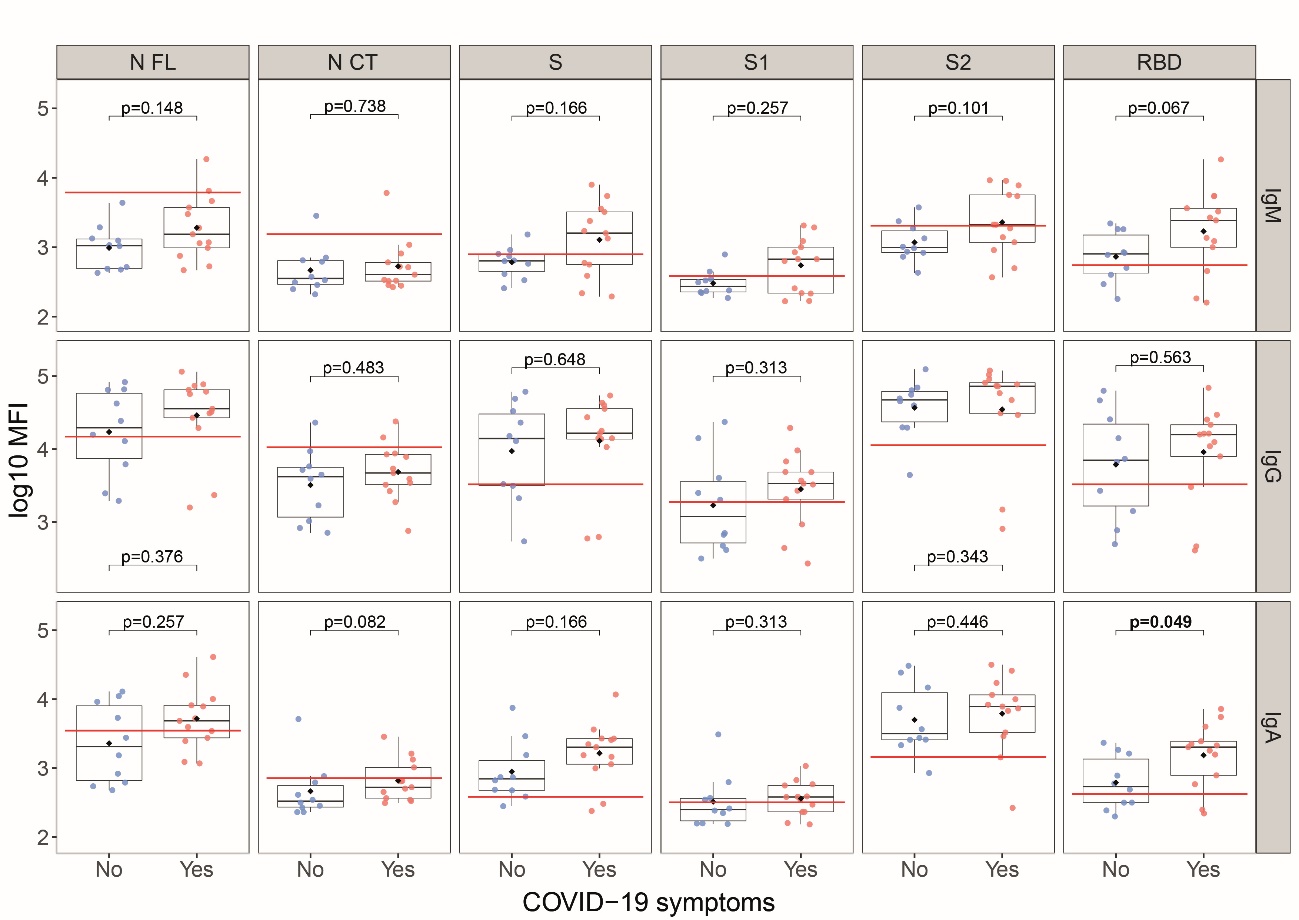
****Figure S1. Anti-SARS-CoV-2 antibody levels in infected mothers by COVID-19 compatible symptoms.** Comparison of anti-SARS-CoV-2 antibody levels (log_10_ median fluorescence intensity, MFI) between mothers with symptomatic (N=13, in red) and asymptomatic COVID-19 (N=10, in blue) during the third trimester. Only infected mothers (positive by rRT-PCR and/or serology) were included in the analysis. The boxplots represent the median (bold line), the mean (black diamond), the 1^st^ and 3^rd^ quartiles (box) and the largest and smallest values within 1.5 times the inter-quartile range (whiskers). Groups were compared by the Wilcoxon*-*rank-sum*-*test*.* The red line indicates the seropositivity cutoff calculated as 10 to the mean plus 3 standard deviations (SD) of log_10_-transformed MFI of 129 pre-pandemic controls. Antigens: nucleocapsid full-length (N FL) and C-terminus (N CT), spike full-length (S), S1 and S2 subunits, and receptor-binding domain (RBD).

**
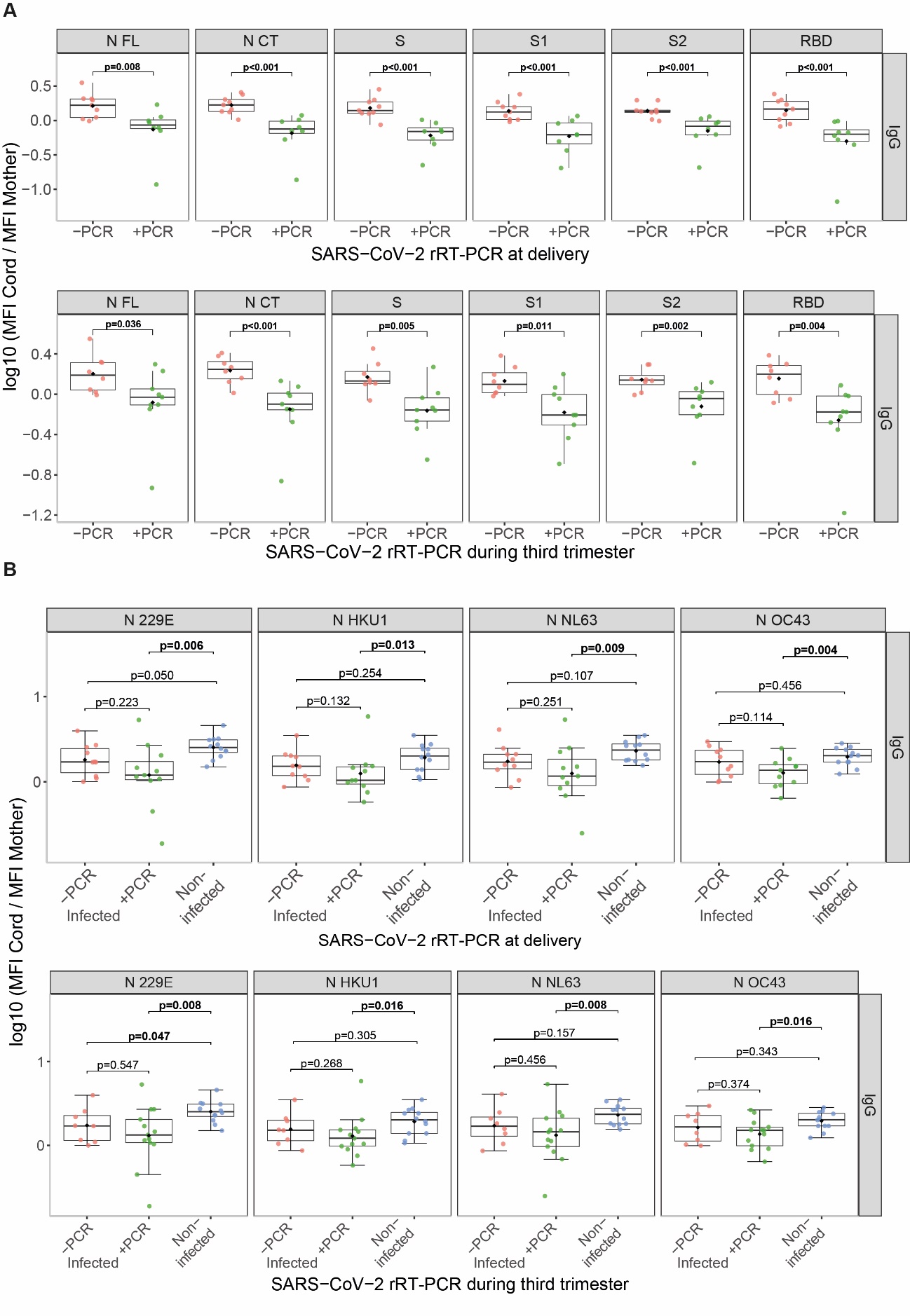
Figure S2. IgG transfer from the mother to the newborn by rRT-PCR status at delivery and at any time during the third trimester. (A)** Comparison of the ratios of IgG levels (log_10_ median fluorescence intensity, MFI) to SARS-CoV-2 antigens in cord blood vs mother peripheral blood from seropositive infected mothers that tested positive by rRT-PCR (+PCR at delivery N=8, third trimester N=9, in green) and negative by rRT-PCR (-PCR at delivery N=9, third trimester N=8, in red). **(B)** Comparison of the ratios of IgG levels (log_10_ MFI) to HuCoVs N protein in cord blood vs mother peripheral blood between infected mothers that tested COVID-19 positive by rRT-PCR (+PCR at delivery N=11, third trimester N=13, in green), those that tested negative (-PCR at delivery N=10, third trimester N=8, in red) and non-infected mothers (N=12, in blue). The boxplots represent the median (bold line), the mean (black diamond), the 1^st^ and 3^rd^ quartiles (box) and the largest and smallest values within 1.5 times the inter-quartile range (whiskers). Groups were compared by the Wilcoxon-rank-sum-test. Antigens: nucleocapsid full-length (N FL) and C-terminus (N CT), spike full-length (S), S1 and S2 subunits, and receptor-binding domain (RBD).

**
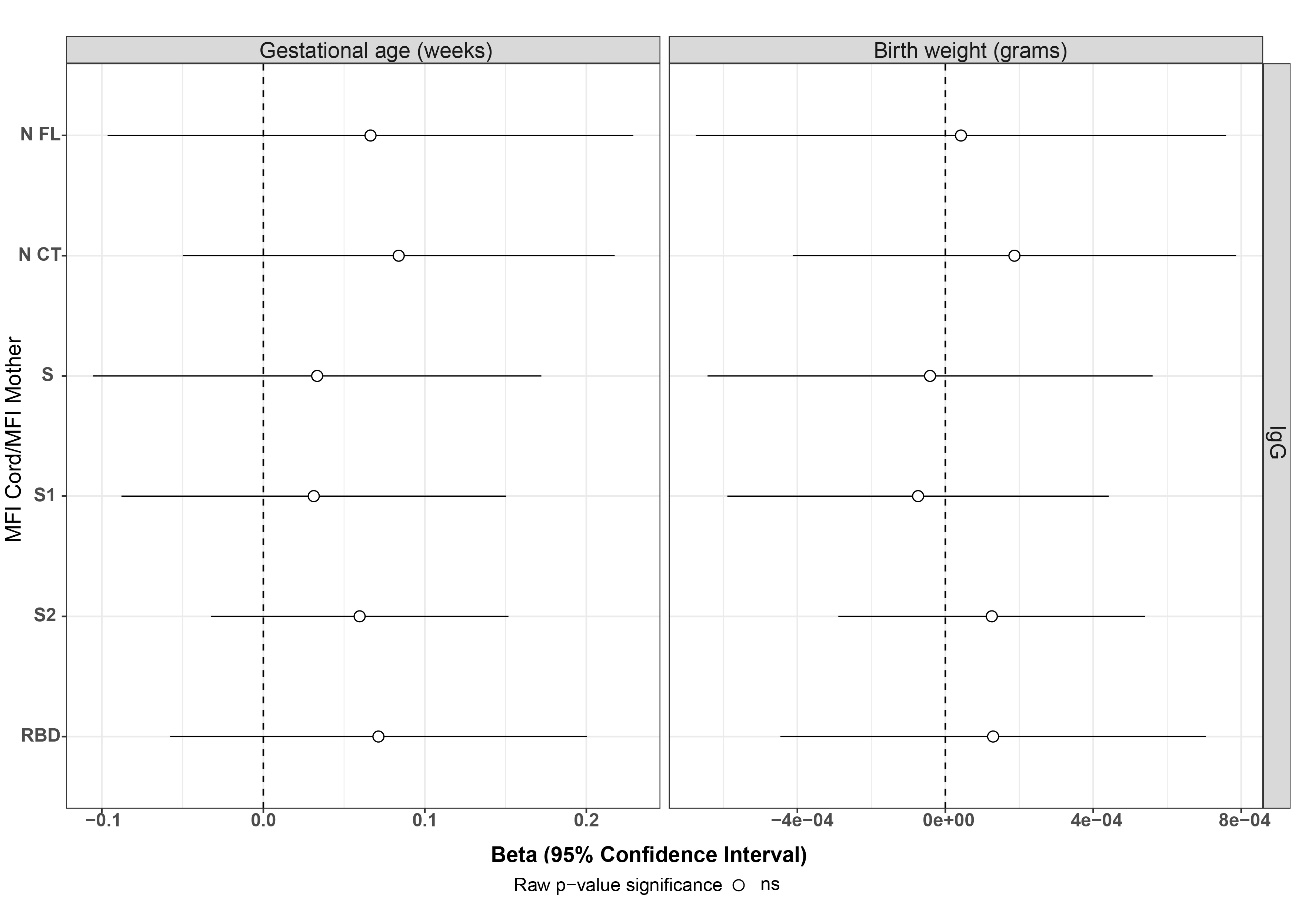
**

**Figure S3. Association of gestational age and birth weight on SARS-CoV-2 IgG transfer from mother to the newborn in univariable linear regression models.** Forest plots show the effect of gestational age, and the birth weight on IgG transfer from mother to the newborn (MFI Cord/MFI Mother) for SARS-CoV-2 antigens in seropositive paired infected mothers and cord blood. Univariable linear regression models were fitted to calculate the betas (dots) and 95% confidence intervals (CI) (lines). The color of the dots represents the p-value significance, where ns= not significant. Antigens: nucleocapsid full-length (N FL) and C-terminus (N CT), spike full-length (S), S1 and S2 subunits, and receptor-binding domain (RBD).


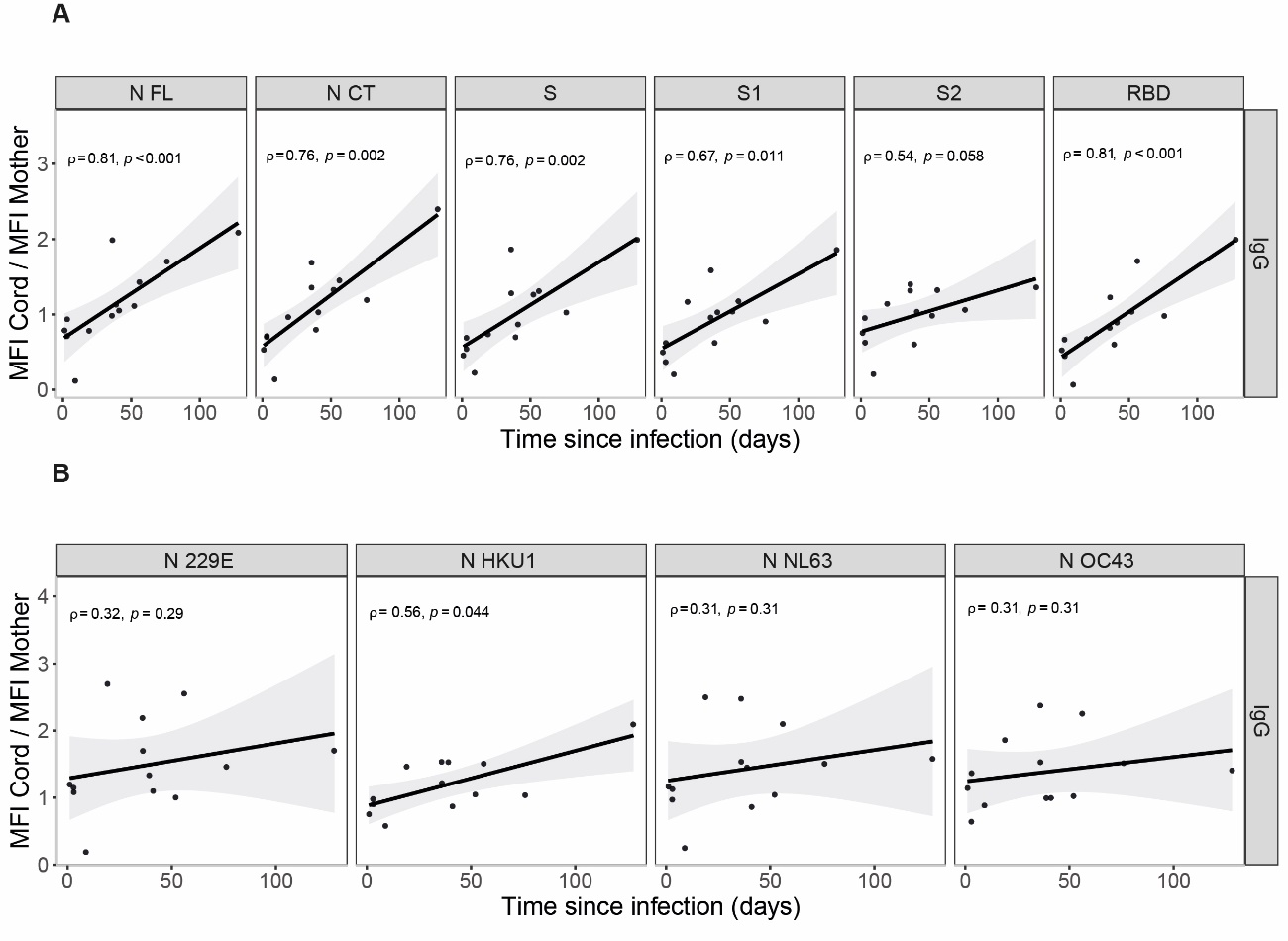
**Figure S4. Correlations between IgG transplacental transfer and time since infection.** Correlations of IgG transplacental transfer (median fluorescence intensity (MFI) Cord/ MFI Mother) of **(A)** SARS-CoV-2 antigens and **B)** HuCoVs antigens in seropositive infected mothers with time since infection days since positive rRT-PCR or symptoms onset), represented as a linear model with standard error as confidence interval (shaded areas) and assessed by the Spearman test, showing the rho (ρ) and p-values. Antigens: nucleocapsid full-length (N FL) and C-terminus (N CT), spike full-length (S), S1 and S2 subunits, and receptor-binding domain (RBD).

**
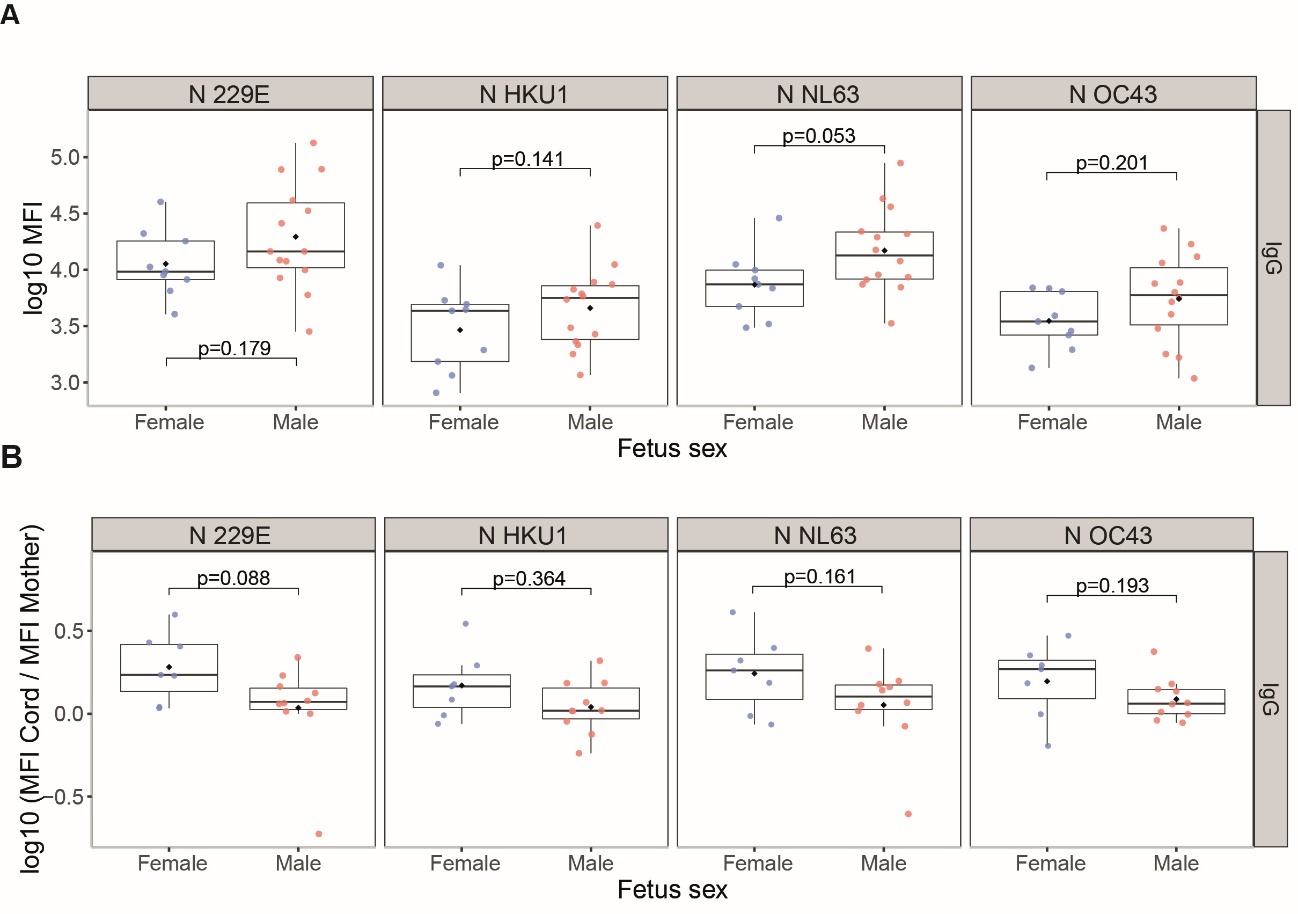
Figure S5. Anti-HuCoVs antibody levels by fetus sex in SARS-CoV-2 infected mothers. (A)** Comparison of anti-HuCoVs levels (log_10_ median fluorescence intensity, MFI) in SARS-CoV-2 infected mothers between those who had a male fetus (N=14, in red) and those who had a female fetus (N=9, in blue). **(B)** Comparison of the ratios of IgG levels (log_10_ MFI) to HuCoVs antigens in cord blood vs mother peripheral blood from SARS-CoV-2 seropositive mothers that had a male fetus (N=10, in red) and those that had a female fetus (N=7, in blue). The boxplots represent the median (bold line), the mean (black diamond), the 1^st^ and 3^rd^ quartiles (box) and the largest and smallest values within 1.5 times the inter-quartile range (whiskers). Groups were compared by the Wilcoxon-rank-sum-test. The red line indicates the seropositivity cutoff calculated as 10 to the mean plus 3 standard deviations (SD) of log_10_-transformed MFI of 129 pre-pandemic controls. Antigens: nucleocapsid full-length (N FL) and C-terminus (N CT), spike full-length (S), S1 and S2 subunits, and receptor-binding domain (RBD).


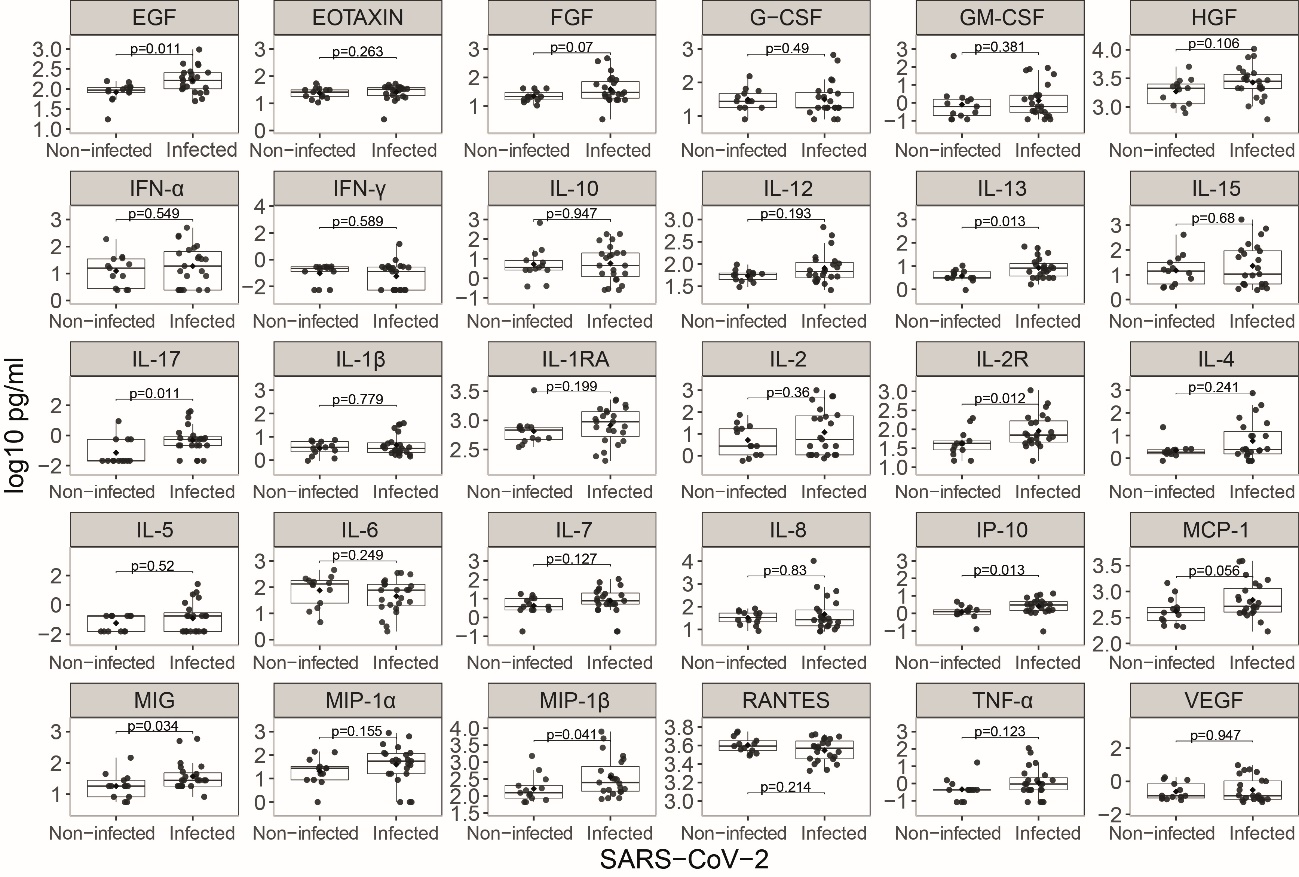
**Figure S6. Cytokine, chemokine and growth factor concentrations in SARS-CoV-2 infected vs non-infected mothers.** Comparison of inflammatory markers concentrations (log_10_ pg/ml) in serum samples between SARS-CoV-2 infected (N=23) and non-infected (N=13) mothers. The boxplots represent the median (bold line), the mean (black diamond), the 1^st^ and 3^rd^ quartiles (box) and the largest and smallest values within 1.5 times the inter-quartile range (whiskers). Groups were compared by the Wilcoxon*-*rank-sum*-*test.

**
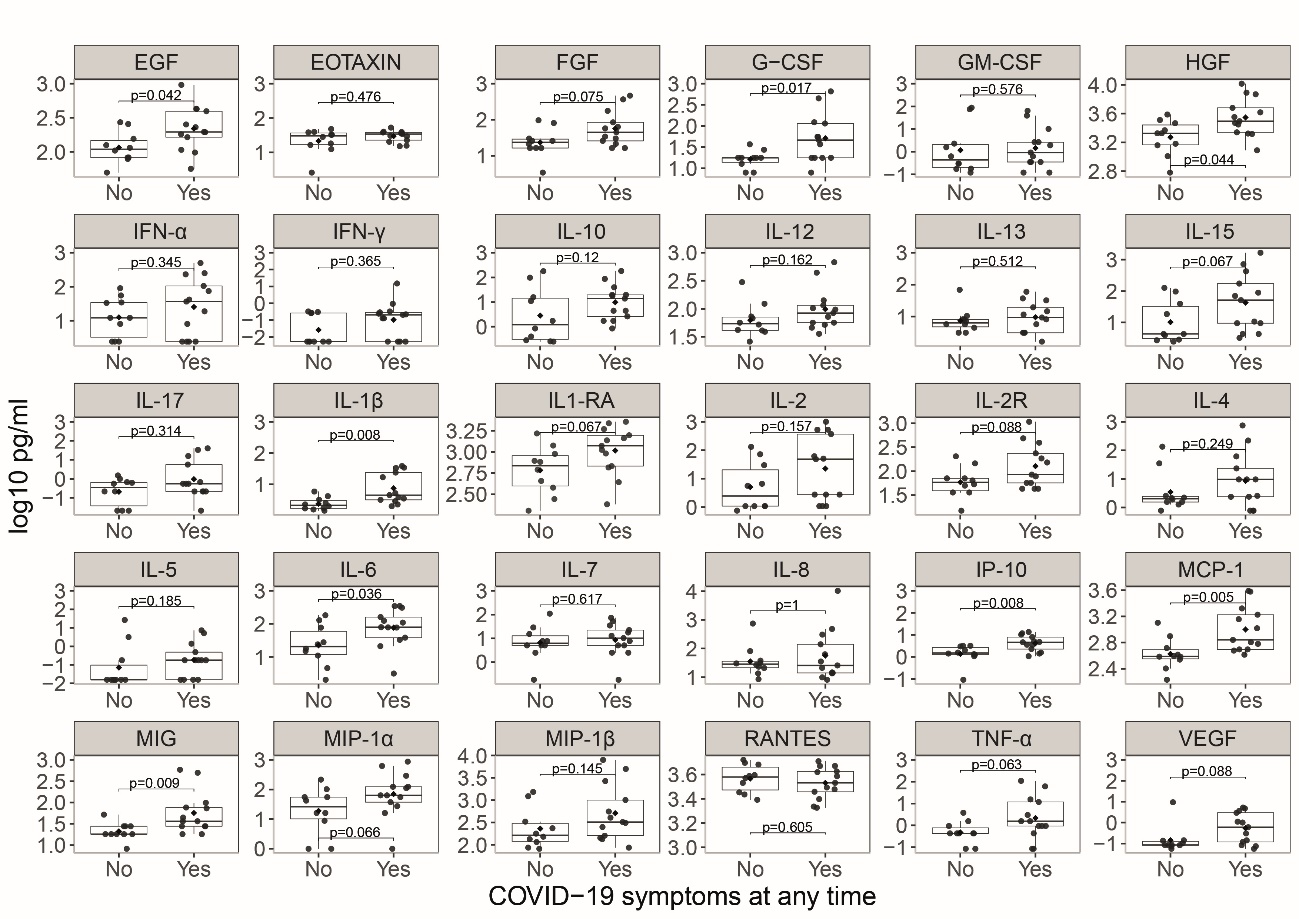
Figure S7. Cytokine, chemokine and growth factor concentrations in SARS-CoV-2 infected mothers symptomatic at any time during the third trimester vs the asymptomatic.** Comparison of inflammatory markers concentrations (log_10_ pg/ml) in serum between infected symptomatic (N=13) and asymptomatic (N=10) mothers during the third trimester. The boxplots represent the median (bold line), the mean (black diamond), the 1^st^ and 3^rd^ quartiles (box) and the largest and smallest values within 1.5 times the inter-quartile range (whiskers). Groups were compared by the Wilcoxon*-*rank-sum*-*test.

**
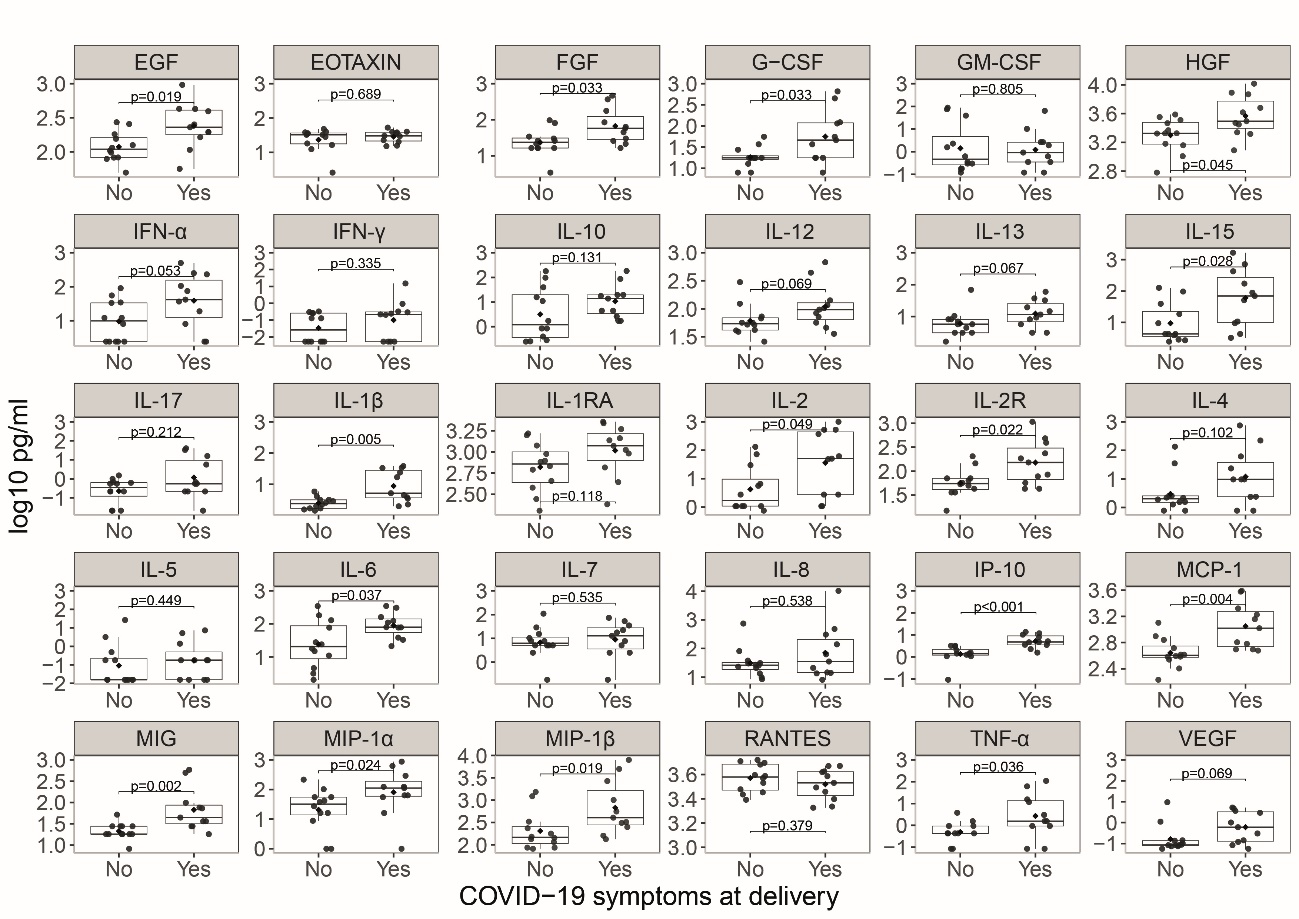
Figure S8. Cytokine, chemokine and growth factor concentrations in SARS-CoV-2 infected mothers symptomatic at delivery vs asymptomatic.** Comparison of inflammatory markers concentrations (log_10_ pg/ml) in serum between infected symptomatic (N=11) and asymptomatic (N=10) mothers at delivery. The boxplots represent the median (bold line), the mean (black diamond), the 1^st^ and 3^rd^ quartiles (box) and the largest and smallest values within 1.5 times the inter-quartile range (whiskers). Groups were compared by the Wilcoxon*-*rank-sum*-*test.

**
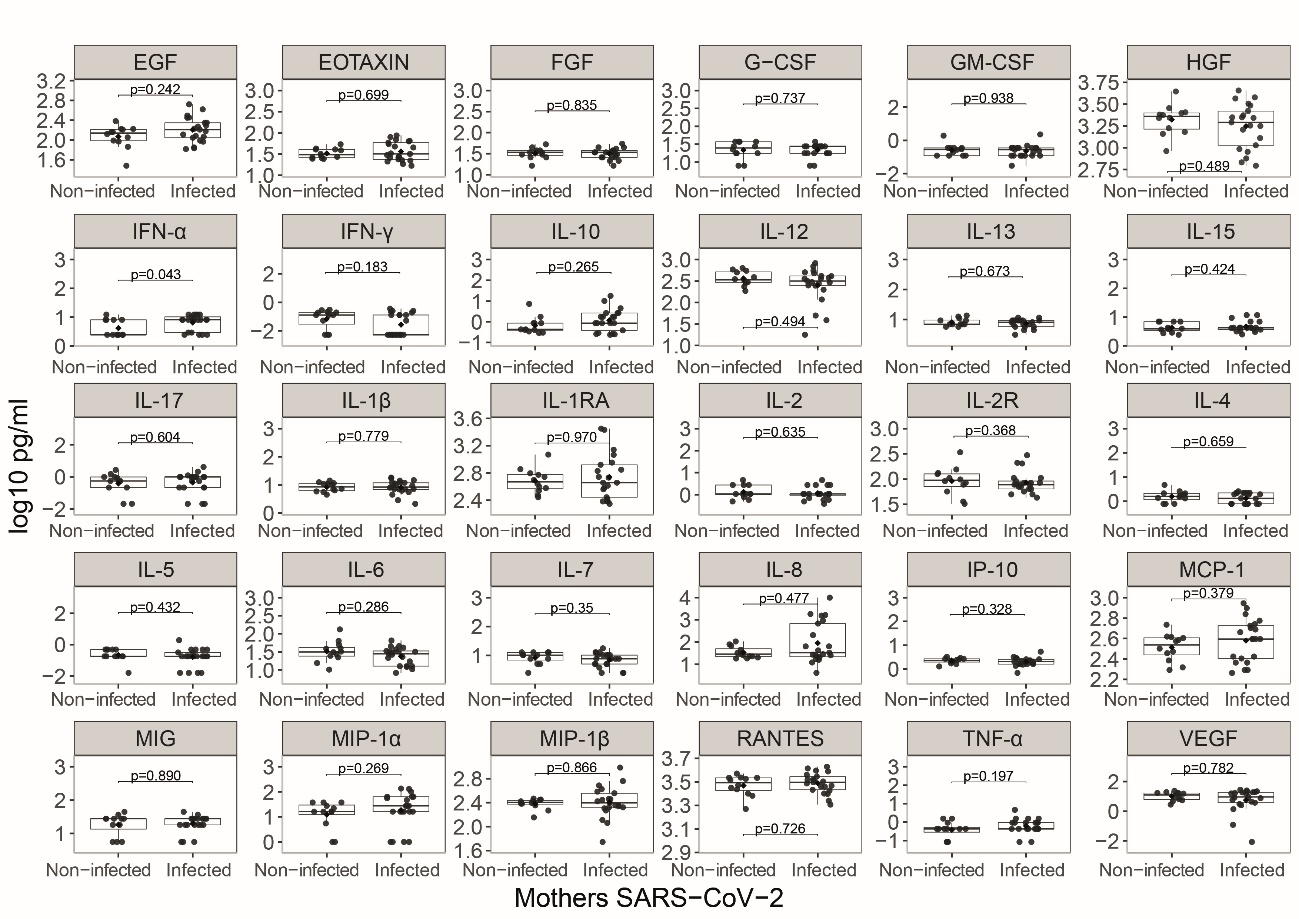
Figure S9. Cytokine, chemokine and growth factor concentrations in cord blood from SARS-CoV-2 infected vs non-infected mothers.** Comparison of inflammatory markers concentrations (log_10_ pg/ml) in cord blood samples between those born from SARS-CoV-2 infected (N=21) and non-infected (N=12) mothers. The boxplots represent the median (bold line), the mean (black diamond), the 1^st^ and 3^rd^ quartiles (box) and the largest and smallest values within 1.5 times the inter-quartile range (whiskers). Groups were compared by the Wilcoxon*-*rank-sum*-*test.


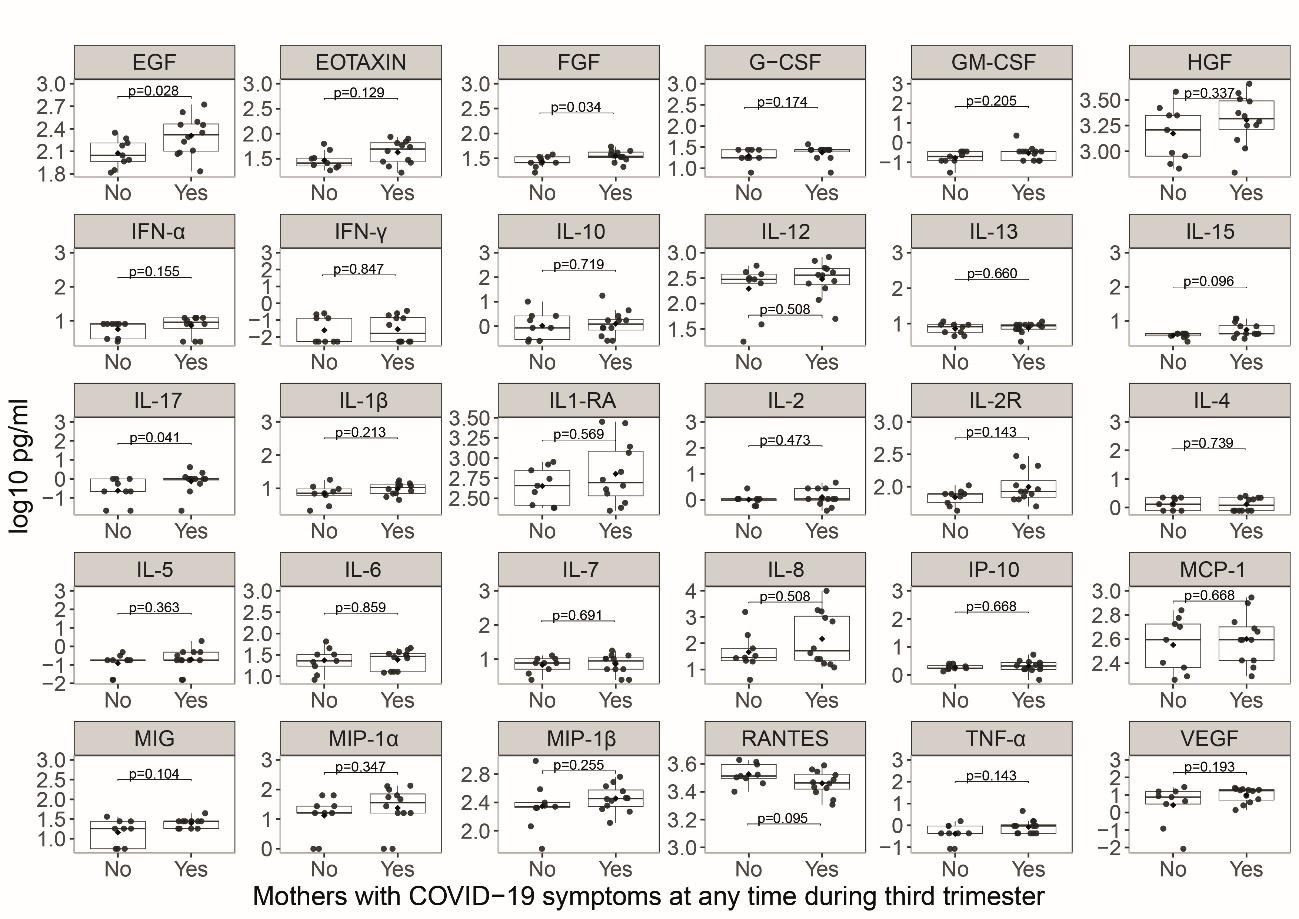
**Figure S10. Cytokine, chemokine and growth factor concentrations in cord blood from SARS-CoV-2 infected mothers symptomatic at any time during the third trimester vs the asymptomatic.** Comparison of inflammatory markers concentrations (log_10_ pg/ml) in cord blood samples from infected symptomatic (N=12) vs asymptomatic (N=9) mothers during the third trimester. The boxplots represent the median (bold line), the mean (black diamond), the 1^st^ and 3^rd^ quartiles (box) and the largest and smallest values within 1.5 times the inter-quartile range (whiskers). Groups were compared by the Wilcoxon*-*rank-sum*-*test.

**
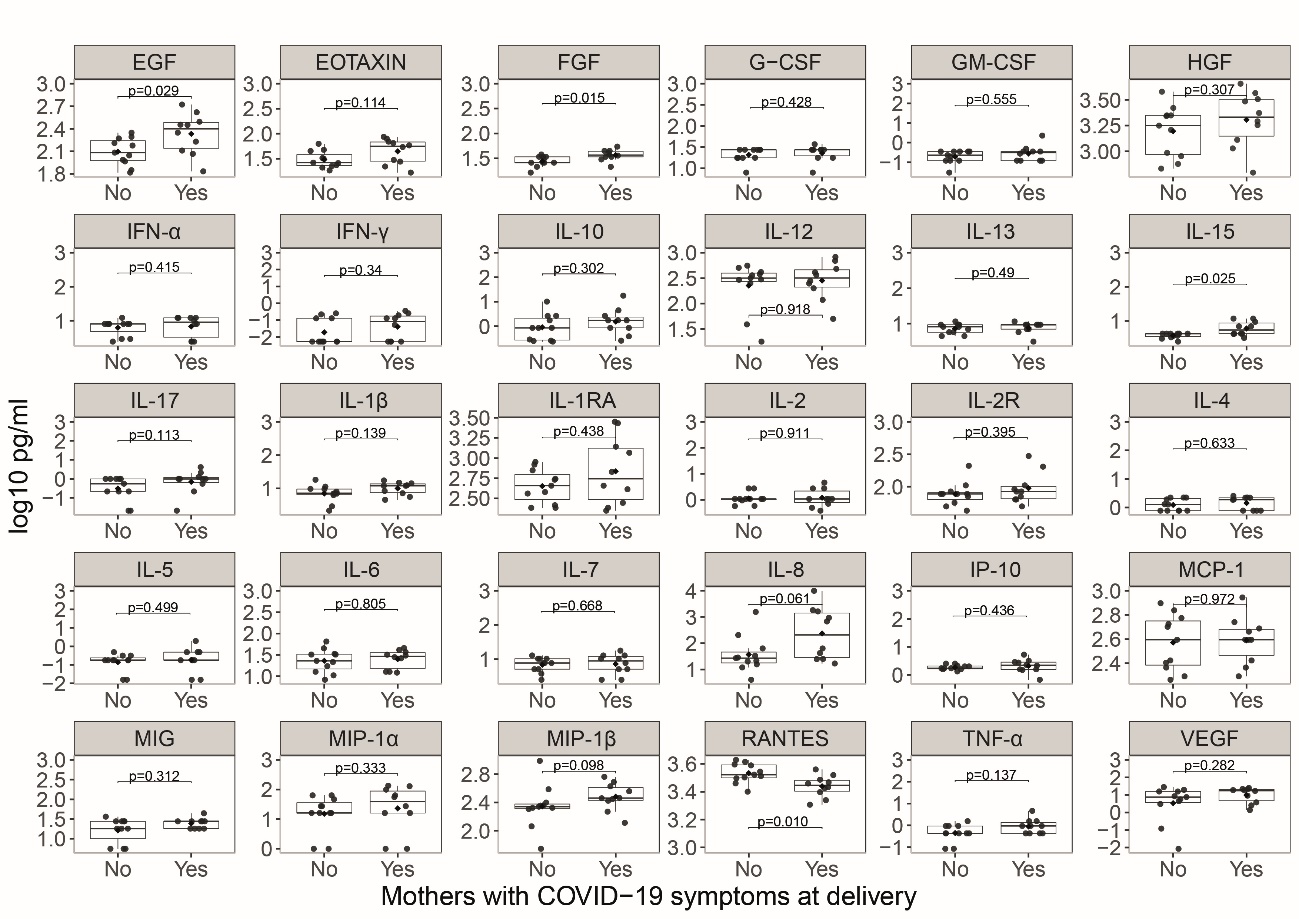
Figure S11. Cytokine, chemokine and growth factor concentrations in cord blood from SARS-CoV-2 infected mothers symptomatic at delivery vs asymptomatic.** Comparison of inflammatory markers concentrations (log_10_ pg/ml) in cord blood samples from infected symptomatic (N=10) vs asymptomatic (N=11) mothers at delivery. The boxplots represent the median (bold line), the mean (black diamond), the 1^st^ and 3^rd^ quartiles (box) and the largest and smallest values within 1.5 times the inter-quartile range (whiskers). Groups were compared by the Wilcoxon*-*rank-sum*-*test.


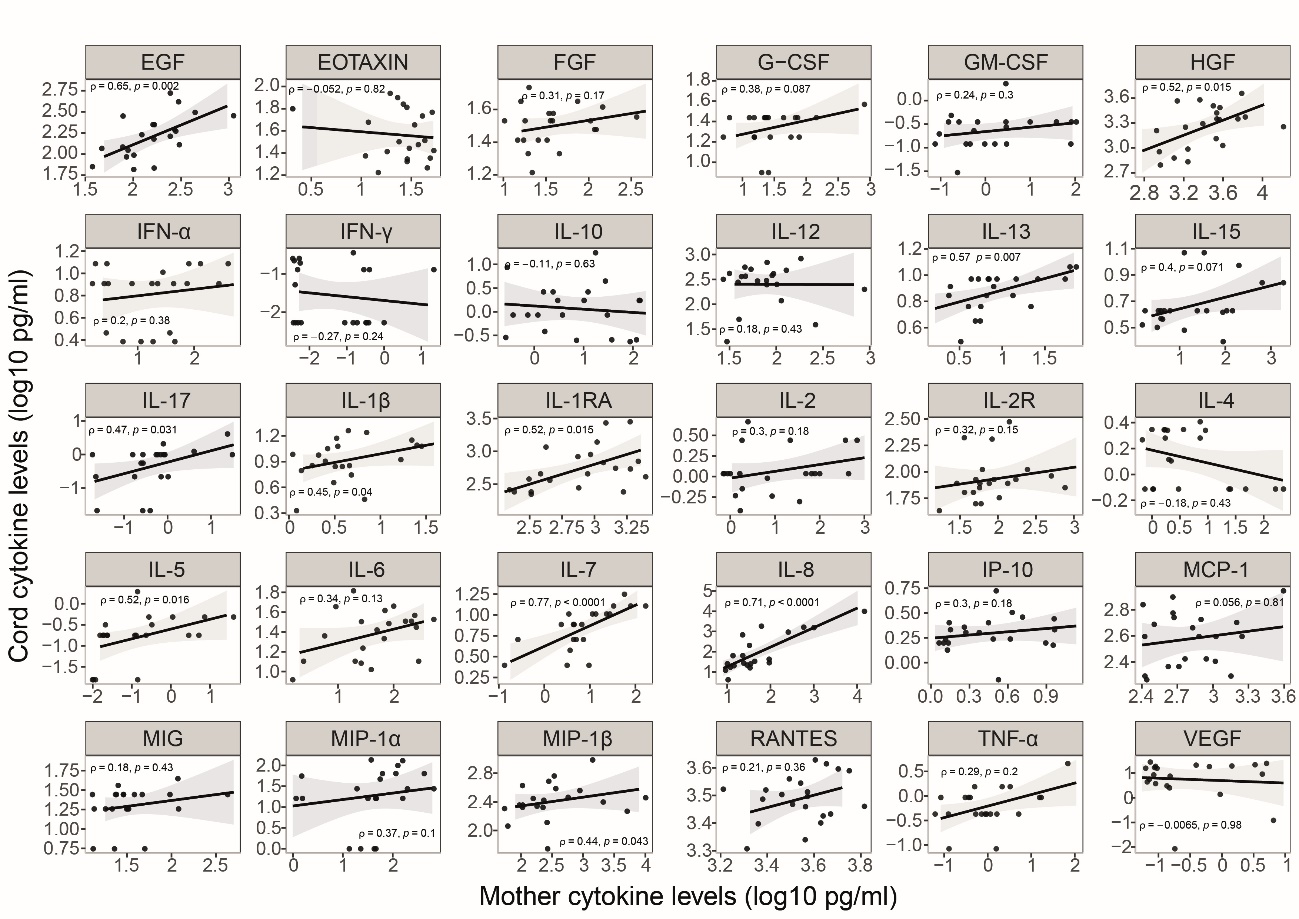
**Figure S12. Correlations of cytokine, chemokine and growth factor concentrations in plasma and cord blood from SARS-CoV-2 infected mothers.** Correlations of inflammatory markers concentrations (log_10_ pg/ml) in serum blood and cord blood from SARS-CoV-2 infected mothers are represented as a linear model with standard error as the confidence interval. Correlations were assessed by the Spearman test, and the rho (ρ) and p-values are shown.

**
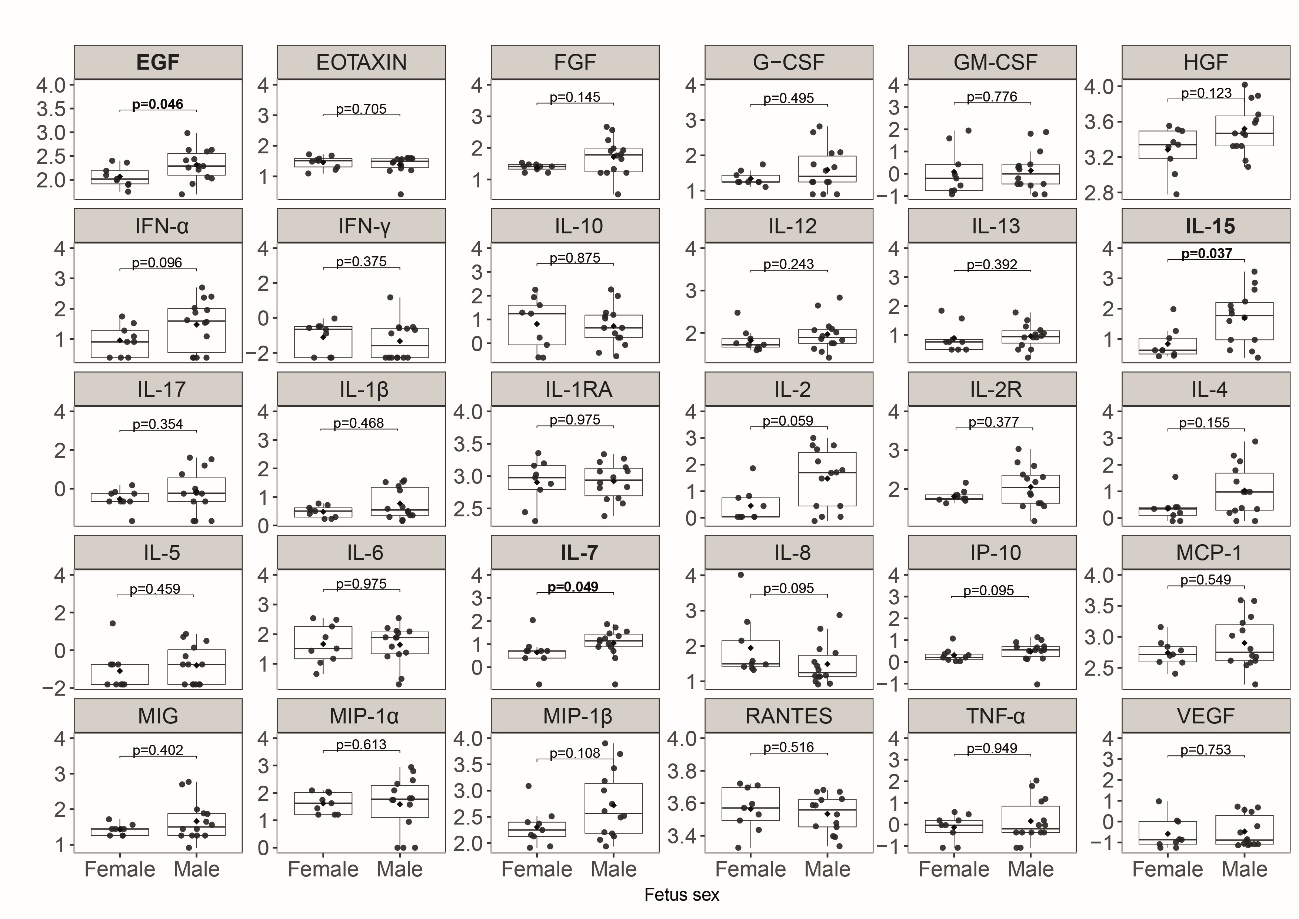
Figure S13. Cytokine, chemokine and growth factor concentrations in SARS-CoV-2 infected mothers by fetus sex.** Comparison of inflammatory markers concentrations (log_10_ pg/ml) in serum from infected mothers between those who had a male fetus (N=14) and those who had a female fetus (N=9). The boxplots represent the median (bold line), the mean (black diamond), the 1^st^ and 3^rd^ quartiles (box) and the largest and smallest values within 1.5 times the inter-quartile range (whiskers). Groups were compared by the Wilcoxon*-*rank-sum*-*test.

# References

1. Dobaño C, Ramírez-Morros A, Alonso S, Vidal-Alaball J, Ruiz-Olalla G, Vidal M, et al. Persistence and baseline determinants of seropositivity and reinfection rates in  health care workers up to 12.5 months after COVID-19. BMC medicine. 2021 Jun;19(1):155.

2. Dobaño C, Santano R, Jiménez A, Vidal M, Chi J, Rodrigo Melero N, et al. Immunogenicity and crossreactivity of antibodies to the nucleocapsid protein of SARS-CoV-2: utility and limitations in seroprevalence and immunity studies. Translational research. 2021 Feb;S1931-5244(21)00029-3.

3. Aguilar R, Campo JJ, Chicuecue S, Cisteró P, Català A, Luis L, et al. Changing plasma cytokine, chemokine and growth factor profiles upon differing malaria transmission intensities. Malaria Journal. 2019;doi:10.1186/s12936-019-3038-x.

4. Ballart C, Torrico MC, Vidal G, Torrico F, Lozano D, Gállego M, et al. Clinical and immunological characteristics of tegumentary leishmaniasis cases in  Bolivia. PLoS neglected tropical diseases. 2021 Mar;15(3):e0009223.

5. Natama HM, Moncunill G, Rovira-Vallbona E, Sanz H, Sorgho H, Aguilar R, et al. Modulation of innate immune responses at birth by prenatal malaria exposure and  association with malaria risk during the first year of life. BMC medicine. 2018 Nov;16(1):198.

6. Sanz H, Aponte JJ, Harezlak J, Dong Y, Ayestaran A, Nhabomba A, et al. drLumi: An open-source package to manage data, calibrate, and conduct quality  control of multiplex bead-based immunoassays data analysis. PloS one. 2017;12(11):e0187901.
